# Generation of human blastocyst-like structures from pluripotent stem cells

**DOI:** 10.1038/s41421-021-00316-8

**Published:** 2021-09-07

**Authors:** Yong Fan, Zheying Min, Samhan Alsolami, Zhenglai Ma, E. Zhang, Wei Chen, Ke Zhong, Wendi Pei, Xiangjin Kang, Puyao Zhang, Yongliang Wang, Yingying Zhang, Linfeng Zhan, Haiying Zhu, Chenrui An, Rong Li, Jie Qiao, Tao Tan, Mo Li, Yang Yu

**Affiliations:** 1 Department of Gynecology and Obstetrics, Key Laboratory for Major Obstetric Diseases of Guangdong Province, Key Laboratory of Reproduction and Genetics of Guangdong Higher Education Institutes, The Third Affiliated Hospital of Guangzhou Medical University, Guangzhou, Guangdong China; 2 Beijing Key Laboratory of Reproductive Endocrinology and Assisted Reproductive Technology and Key Laboratory of Assisted Reproduction, Ministry of Education, Center of Reproductive Medicine, Department of Obstetrics and Gynecology, Peking University Third Hospital, Beijing, China; 3 Biological and Environmental Sciences and Engineering Division, King Abdullah University of Science and Technology (KAUST), Thuwal, Kingdom of Saudi Arabia; 4 Yunnan Key Laboratory of Primate Biomedical Research, Institute of Primate Translational Medicine, Kunming University of Science and Technology, Kunming, Yunnan China; 5 School of Mechanics and Civil Engineering, China University of Mining and Technology, Beijing, China; 6 Clinical Stem Cell Research Center, Peking University Third Hospital, Beijing, China

**Keywords:** Embryonic stem cells, Pluripotency

## Abstract

Human blastocysts are comprised of the first three cell lineages of the embryo: trophectoderm, epiblast and primitive endoderm, all of which are essential for early development and organ formation. However, due to ethical concerns and restricted access to human blastocysts, a comprehensive understanding of early human embryogenesis is still lacking. To bridge this knowledge gap, a reliable model system that recapitulates early stages of human embryogenesis is needed. Here we developed a three-dimensional (3D), two-step induction protocol for generating blastocyst-like structures (EPS-blastoids) from human extended pluripotent stem (EPS) cells. Morphological and single-cell transcriptomic analyses revealed that EPS-blastoids contain key cell lineages and are transcriptionally similar to human blastocysts. Furthermore, EPS-blastoids are similar with human embryos that were cultured for 8 or 10 days in vitro, in terms of embryonic structures, cell lineages and transcriptomic profiles. In conclusion, we developed a scalable system to mimic human blastocyst development, which can potentially facilitate the study of early implantation failure that induced by developmental defects at early stage.

## Introduction

Human embryogenesis begins with a fertilized egg and then undergoes cell divisions, lineage segregations, and morphogenic rearrangements lay the foundation for blastocyst formation. Following activation of the embryonic genome and the beginning of compaction and polarization, blastomeres undergo lineage segregation and morphogenetic rearrangements to form a ball-shaped structure termed the blastocyst^1–3^. Blastocysts contain specialized cell types, namely epiblast (EPI), primitive endoderm (PE), and trophectoderm (TE)^4–6^. In recent years, “multi-omic” technologies have enabled researchers to chart the gene-transcription and chromatin-modification landscapes of these cell types, providing valuable information regarding human embryogenesis^7–10^. However, the supply of human embryos is extremely limited due to ethical and technical limitations, thereby precluding a precise mechanistic understanding of early human embryogenesis. To systematically interrogate the early human development, a robust in vitro model of human embryogenesis is urgently needed.

Using human embryonic stem cells (hESCs), researchers have been working toward modeling embryogenesis in a dish. Previous studies have reported that hESCs cultured in three-dimensional (3D) soft-gel or a microfluidics device can form embryonic sac-like structures that mimic the early postimplantation human EPI and amnion development^11–13^. Recently, 3D human gastrulating embryo-like structures (gastruloids) were generated by subjecting hESCs to a pulse of Wnt agonist, allowing modeling of the spatiotemporal organization of the three germ layers during gastrulation^14^. However, all these models use only EPI-derived hESCs and lack cells resembling the TE and PE. Therefore, they can not fully recapitulate the lineage interactions that characterize human embryonic development. In addition, recent studies suggested that hESCs are of postimplantation EPI origin and represent the primed pluripotency state^5,15^. Therefore, hESCs may not be suitable for modeling preimplantation development.

Recently, researchers have attempted to generate human stem cells resembling those in the preimplantation embryo (i.e., exhibiting naive pluripotency). These efforts revealed a continuum of pluripotency in the form of human naive stem cells or extended pluripotency stem cells (EPS cells)^16,17^. With these cells, it became possible to test whether they can self-organize into preimplantation embryo-like structures. This interest was further stimulated by recent success in generating mouse blastocyst-like structures, blastoids^18–20^. Mouse EPS cell aggregates recapitulate several morphogenic hallmarks of preimplantation embryogenesis and differentiate into both embryonic and extra-embryonic lineages, thus forming blastoids that share many features of the blastocyst. Recently, three groups have reported the reprogramming of fibroblasts into in vitro three-dimensional models of the human blastocyst, and the generation of blastocyst-like structures in vitro from naive human pluripotent stem cells^21–23^. However, given the significant differences between mice and humans, it is unclear whether human EPS cells can also generate blastoids in vitro to mimic human embryogenesis from preimplantation to postimplantation stages.

In this study, we developed a 3D, two-step differentiation protocol for generating human blastocyst-like structures from human EPS cells, named EPS-blastoids. Human EPS-blastoids partially resembled human blastocyst in terms of morphology, specific markers for the three cell lineages, and global transcriptome signatures at single-cell resolution. Importantly, further in vitro culturing of these human EPS-blastoids resulted in the emergence of structures similar to those observed in early postimplantation embryos.

## Results

### A 3D two-step differentiation method for generating human blastoid

By using an established protocol^16^, we converted human induced pluripotent stem cells (iPSCs) into EPS cells^16^. Then we attempted to generate human blastoids from these EPS cells using a modified protocol for generating mouse blastoids^18^. However, EPS cell aggregates treated with induction media containing BMP4 generally failed to form a cavity-containing structure after 5 days of induction (Supplementary Fig. S1a). A few aggregates appeared to contain a small cavity, but they were enclosed by a membrane instead of TE-like cells and did not have the typical morphology and size of a blastocyst. Immunofluorescence labeling showed that the majority of these solid structures expressed the EPI marker OCT4 and the PE marker GATA6, while small fraction (~10% of day 6 aggregates) also expressed the TE markers CK8 and GATA2/3 (about 30%–50% of TE cells in each aggregate), albeit partially (Supplementary Fig. S1b, c). These results indicate that at least a small number of human EPS cells were capable of differentiating into the TE lineage with BMP4 induction using the modified mouse blastoids protocol^18^.

We then attempted to pretreat human EPS cells with BMP4 to generate TE-like cells. First, gene expression was analyzed via real-time quantitative PCR (qPCR) during a time-course of BMP4 induction to optimize human EPS cell differentiation conditions. Between day 0 and day 5 of BMP4 stimulation, EPI-specific genes were gradually downregulated as TE-specific genes were upregulated (Supplementary Fig. S1d). Interestingly, genes characteristic of mid- and late-TE were activated by BMP4 induction, with most TE-specific genes reaching 1.5–10-fold induction by day 3 (30- and 500-fold for *WNT7* and *GATA3*, respectively) (Supplementary Fig. S1e). Higher levels of induction were observed on day 5 of BMP4 treatment. TE-like cells exhibited morphological changes by 2 days of BMP4 induction, with cells becoming flattened and enlarged (Supplementary Fig. S1f). Immunostaining revealed more GATA2/3-, CK7-, CK8-, and TFAP2C-positive cells on day 3 compared with day 1 or 2 (Supplementary Fig. S1g, h). As day 3 marks the onset of significant gene expression and morphological changes, we decided to use TE-like cells subjected to 3 days of BMP4 pretreatment for subsequent experiments.

Given the importance of TE-like cells for making blastoids structures, we further characterized these EPS-derived TE-like cells and compared them to TE derivatives of pluripotent stem cells (PSCs). We performed RNA sequence (RNA-seq) analysis of EPS-derived TE-like cells following BMP4 or BAP (BMP4, A83-01, and PD0325901) treatments and compared the gene expression data with published data of TE-like cells derived from human naive PSCs treated with BAP or with A83-01 and PD 0325901 (PDA83)^24,25^. Human EPS cells and naive PSCs clustered together in principal component analysis (PCA), while TE-like cells formed a separate cluster. EPS cells and naive PSCs shared a similar differentiation trajectory when subjected to TE-inducing conditions (Supplementary Fig. S2a). qPCR analysis showed that genes associated with TE, such as GATA3, TFAP2C, GATA2, DAB2, and KRT18, which was also observed in BMP4-treated naive PSCs, were significantly upregulated in EPS cells treated with BMP4 for 3 days (Supplementary Fig. S2b). Moreover, hierarchical clustering of gene expression showed that while EPS-derived TE-like cells had a comparable (hEPS-BMP/BAP vs PXGL_PDA83) or better (hEPS-BMP/BAP vs iNPSC_BAP) upregulation of TE marker genes when compared to naive PSCs TE derivatives, they extinguished the pluripotency program more completely (Supplementary Fig. S2c). On the other hand, EPS cells expressed high levels of pluripotency genes associated with the preimplantation naive state (e.g., KLF4 and NANOG) but not the postimplantation primed state (e.g., ZIC2), and efficiently downregulated pluripotency genes upon BMP4 treatment (Supplementary Fig. S2b, d). These data suggest exposure of EPS cells to BMP4 for 3 days enabled reliable TE fate transition.

**Fig. 1:**
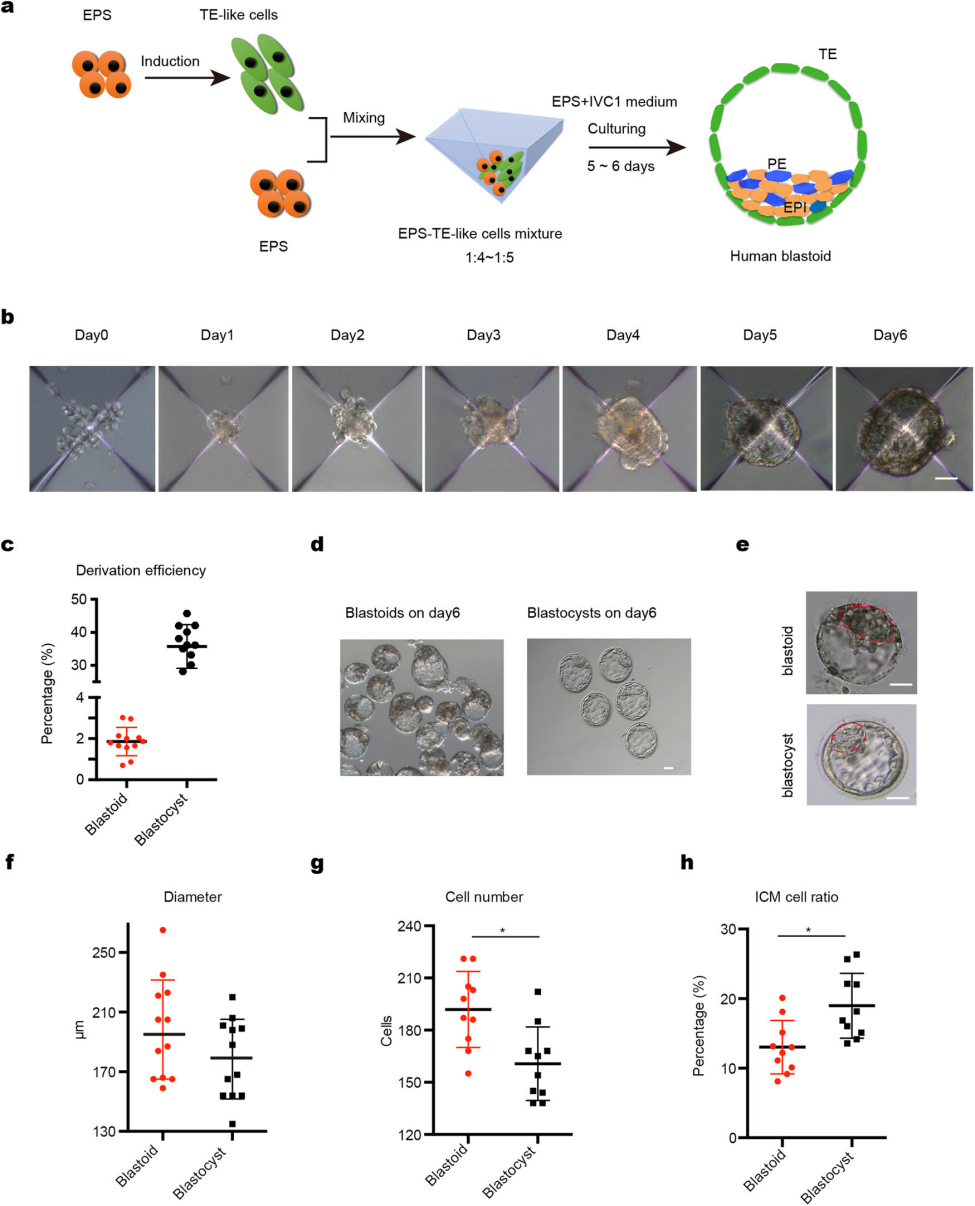
Induction of human blastoids under 3D two-step condition. **a** Schematic of human blastoid formation. EPS cells were firstly induced to TE-like cells, and then TE-like cells were mixed with EPS cells and seeded together to AgreeWell on day 0. The aggregates further differentiated and organized into a human EPS-blastoid. **b** Phase-contrast images of human aggregates in the indicated days showing the formation of human blastoids from day 0 to day 6. Scale bar = 5 μm. **c** Derivation efficiency of human blastoids is about 1.9% that significantly lower than the developmental efficiency of human blastocysts. **d** Phase-contrast image of human blastoids on day 6, Scale bar = 50 μm. **e** Phase-contrast image of human EPS-blastoid (upper) and human blastocyst (lower). Red line indicated inner cell mass (ICM) of the structure and the outer layer cells represented trophoblast cells (TE). Scale bar = 50 μm. **f**–**h** Mean diameter (**f**), total cell number (**g**), and ICM cell ratio (**h**) were quantified between human EPS-blastoids and blastocyst. *n* = 30 EPS-blastoids, *n* = 30 blastocyst. Data in **c**, data are means ± SD (*n* = 12 blastoids). **P < 0.001. Data in **e**, data are means ± SD (*n* = 12 blastoids). *P* > 0.05. Data in **f** and **g**, data are means ± SD (*n* = 12 blastoids). *P < 0.05. Data in **h**, data are numbers (*n* = 40 blastoids, 40 blastocysts), **P < 0.001.

To generate human blastoids, we first pretreated human EPS cells with BMP4 to generated TE-like cells, and then mixed human EPS cells with these TE-like cells at a ratio of 1:4–1:5 (Fig. 1a). After 24 h, these loosely connected cells formed small aggregates, which grew and formed structures with a small cavity by day 4. By days 5–6, blastocyst-like structures were apparent (Fig. 1b), with about 1.9% exhibiting typical blastocyst morphology (Fig. 1c). Morphologically these human blastoids were similar to natural human blastocysts (Fig. 1d, e) in terms of average diameter, but blastoids seemed to have more total cells and fewer cells in the inner cell mass (ICM) (Fig. 1f–h).

### Human EPS-blastoids contain cells of the three lineages of blastocysts

Mixing TE-like cells and EPS cells resulted in cell aggregation and the formation of blastocyst-like structures (EPS-blastoids) on day 5. To investigate whether EPS-blastoid formation recapitulated key cellular processes, we monitored cellular dynamics of blastoids during days 2 and 3. We found that the cell-adhesion protein, E-cadherin (E-cad), localized to cell–cell junctions, indicating cell–cell interactions and communications within EPS aggregates^26^ (Supplementary Fig. S3a). Ki-67 labeling showed the proliferation of EPS-blastoids on days 5 and 6 (Supplementary Fig. S3b).

**Fig. 2:**
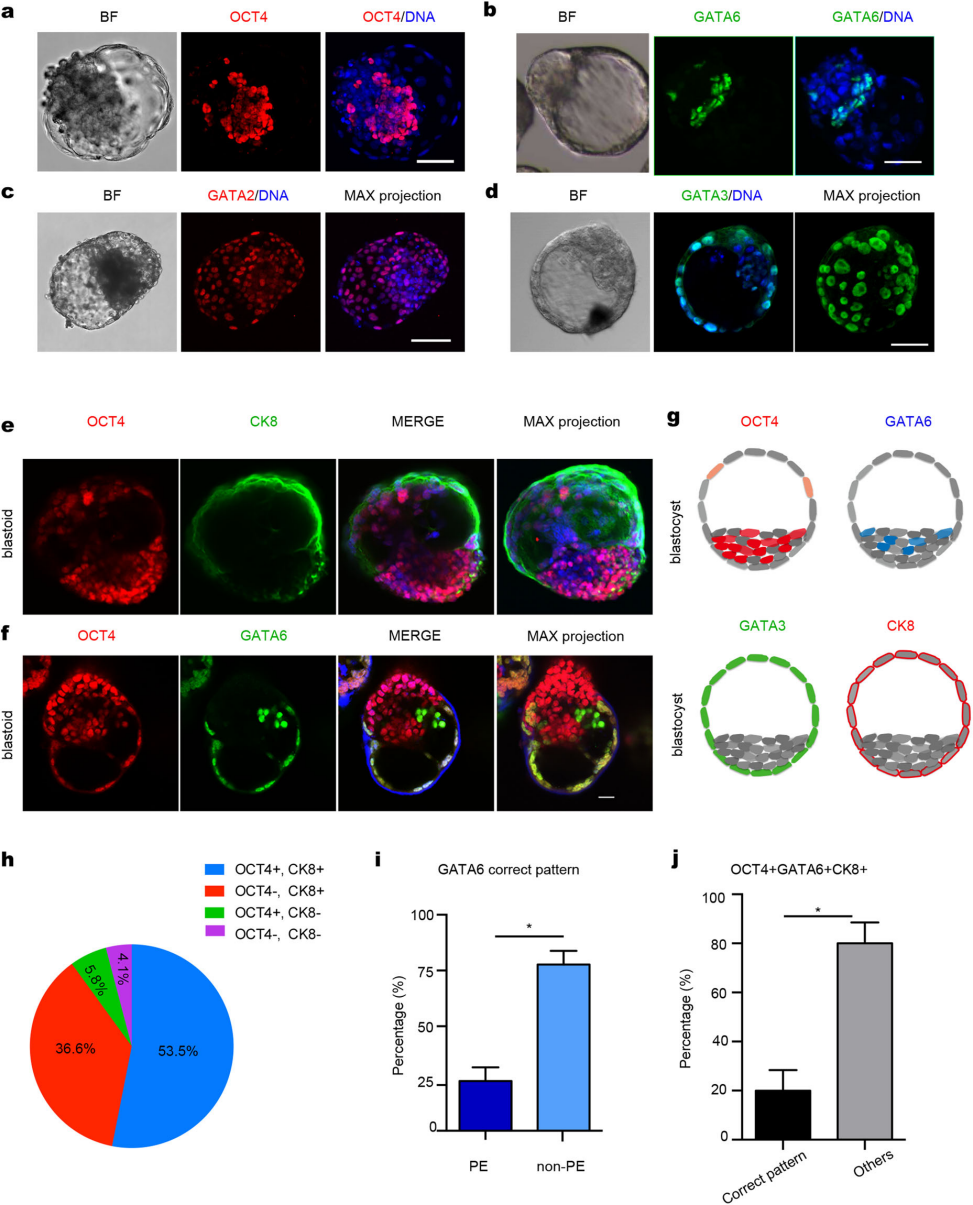
Expression of specific development-related markers. **a**–**f** Immunofluorescence staining of human EPS-blastoids for EPI lineage marker (OCT4), TE lineage markers (CK8 and GATA2/3), and PE lineage marker (GATA6), Scale bar = 50 μm. **g** Model of immunofluorescence staining of human blastocyst for OCT4 in EPI, GATA6 in PE, GATA2/3, and CK8 in TE lineages, Scale bar = 50 μm. **h**–**j** Quantification of the percentage of human blastoids with correct allocation of OCT4 and CK8 (**h**), GATA6 (**i**), and all three markers (**j**). Data in **i**, data are means ± SD (*n* = 172 blastoids). *P < 0.05. Data in **i**, **j**, data are means ± SD (*n* = 131 blastoids).

We sought to determine whether blastoids contained the three cell lineages of the blastocyst, namely EPI, PE and TE, as all three are necessary for an embryo to develop beyond implantation. Immunofluorescence analysis of day 4 blastoids revealed extremely low levels of OCT4 in the outer cell layer (TE cells), with highest levels localized to EPI cells in the interior of the ICM. Cells surrounding these OCT4-positive cells expressed the PE marker, GATA6, as seen in natural early blastocysts^27^ (Supplementary Fig. S4a). Immunofluorescence analysis of EPS-blastoids during days 5 and 6 revealed that OCT4-positive cells localized exclusively to the ICM-like compartment (Fig. 2a, e), whereas cells in the outer layer expressed the TE-specific transcription factors, GATA2 and GATA3 (Fig. 2c, d). The outer layer of cells also expressed the trophoblast (TrB)-specific cytokeratin, KRT8 (CK8), indicative of TE specification (Fig. 2e). The PE marker, GATA6, localized to cells adjacent to the OCT4-positive cells (not those within the outer layer of the blastoids) (Fig. 2b, f). This pattern of localization is similar to that seen in human blastocysts (Fig. 2g). In some blastoids, the positive signal of OCT4 and GATA6 can be detected in TE (Fig. 2e, f), indicating the incomplete programming of TE lineage from EPSCs.

We then calculated the percentage of CK8-positive or OCT4-positive cells in day 6 EPS-blastoids (*n* = 172). These analyses revealed a smaller fraction of OCT4-positive cells than that in human blastocysts, whereas the fraction of CK8-positive cells was reminiscent of human blastocysts. On average, there were ~15% and 80% of cells expressed OCT4 and CK8 in one blastoid, respectively (Supplementary Fig. S3c, d). Of these, 53.5% exhibited the correct pattern of ICM- (OCT4^+^) and TE-like (CK8^+^) localization, 36.6% exhibited only correct TE-like pattern (CK8^+^), 5.8% had only the ICM-like lineage, and 4.1% exhibited mislocalization of ICM- and TE- like cells (Fig. 2h). With further development of early blastocysts, the ICM divides into two lineages: EPI and PE. We examined whether blastoids could develop into late blastocyst-like structures composed of three lineages (EPI, PE and TE). Of 131 blastoids on day 6, about 8% cells of one blastoid were positive for GATA6 expression on average (Supplementary Fig. S3e). Of these, 26% showed PE-like lineage (GATA6^+^, in a stochastic manner) (Fig. 2i), 21% expressed all three lineage markers (Fig. 2j). Moreover, around 80% of the blastoids expressed OCT4, CK8, or GATA6 (Supplementary Fig. S3f–h). Using this induction system, a large proportion of EPS cells failed to form blastoids, although they expressed markers indicative of the EPI, PE and TE lineages. They instead retained features of day 4 aggregates (Supplementary Fig. S4a). In some abnormal day 6 aggregates, OCT4, SOX2 or GATA6 localized improperly to TE cells (Supplementary Fig. S4b–e) instead of the ICM. In summary, these results demonstrated that EPS-blastoids recapitulated the segregation of TE and ICM cell lineages, and that these blastoid structures possessed the three lineages typical of a blastocyst, however, the incomplete programmed EPSCs can be still detected in some blastoids.

To confirm the existence of EPI and TE lineages in EPS-blastoids, we attempted to derive ESCs, PSCs, and trophoblast stem cells (TSCs) from day 6 EPS-blastoids. We were able to establish 3 PSC lines from 6 EPS-blastoids, and 4 TSC lines from 10 EPS-blastoids using the culture condition reported previously^28,29^ (Supplementary Fig. S5a, e). PSCs and TSCs were morphologically similar to those derived from blastocysts (Supplementary Fig. S5b, f). PSC colonies expressed the pluripotency markers OCT4, SOX2, SSEA4, and TRA-1-60 (Supplementary Fig. S5c), whereas TSC colonies expressed the TE-specific markers GATA3, CK7, and TFAP2C (Supplementary Fig. S5g). Further, PSCs derived from EPS-blastoids showed the trilineage differential potential (Supplementary Fig. S5d). TSCs derived from blastoids can generate syncytiotrophoblasts (STBs) and extravillous cytotrophoblasts (EVTs) (Supplementary Fig. S5h–j). The PCA indicated that PSCs and TSCs derived from blastoids showed a closer transcriptional resemblance to the PSCs and TSCs lines^25^ (Supplementary Fig. S5K).

### Single-cell transcriptome analysis of human blastoids

**Fig. 3:**
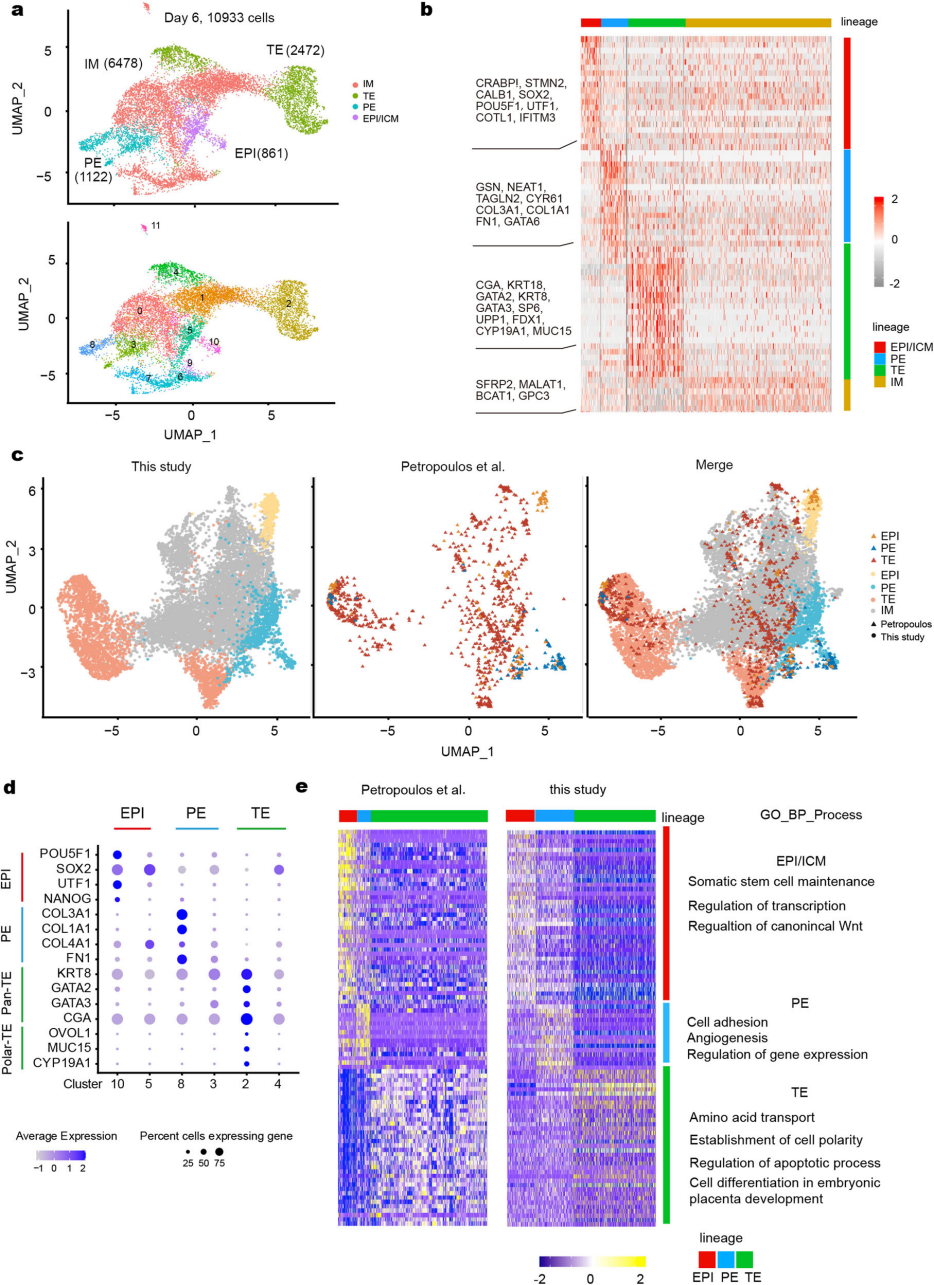
Landscape of transcriptome in human blastoids on day 6. **a** A UMAP plot 10,933 cells from human blastoids showing the cells in blastoids were divided into 4 major clusters on day 6. EPI/ICM, PE, TE, and IM (intermediate) subgroups were determined according to the lineage-specific markers. The cells in IM subgroups express all three lineages markers or some uncertain genes. **b** Heat map of lineage signature genes expression on day 6. **c** UMAP projection of integrated datasets showing EPI-, PE-, and TE-like cells (and IM clusters) together with EPI, PE, and TE cells of blastocysts from a previous published study^7^. **d** Dot plot indicating the markers of EPI, PE, and TE lineage markers. **e** Overlapping genes expression between a previous study^7^ and this study was performed by heat map and GO term analysis.

We performed single-cell RNA-sequencing (scRNA-seq) analysis of 200 day 6 human blastoids. After quality control and filtering, 10,933 single cells were further analyzed using the bioinformatic suite Seurat. Uniform manifold approximation and projection (UMAP) clustering analysis showed that cells from human blastoids could be divided into 12 clusters (Supplementary Fig. S6a). Base on the expression of marker genes (Supplementary Fig. S6a), we characterized two clusters as EPI/ICM, two clusters as PE, and two clusters as TE. The remaining six clusters seemed to express both ICM and TE markers and could each represent an intermediate cell type (Fig. 3a, b). In addition, we performed unsupervised clustering analysis and confirmed that the top genes specific for each lineage were consistent with the UMAP clustering results. The EPI/ICM cluster expressed *OCT4* (*POU5F1*), *NANOG*, and *SOX2*, the PE cluster expressed *FN1*, *COL3A1*, and *GATA6*, and the TE cluster expressed *GATA2* and *GATA3* (Fig. 3b, d; Supplementary Fig. S6c). UMAP analysis showed blastoid EPI-, TE-, and PE-like clusters together with EPI, TE, and PE cells of blastoids from two recent published study (Supplementary Fig. S6d).

To reveal similarities and differences between EPS-blastoids and natural blastocysts, we compared our scRNA-seq data from day 6 EPS-blastoids with a dataset acquired from human blastocysts^7^. Comparisons of our results with data derived from E5–E7 blastocysts^7^ revealed 40, 16, and 37 genes that overlapped with those in EPS-blastoid EPI, PE, and TE clusters, respectively (Supplementary Fig. S6b Table S1). UMAP analysis revealed that cells from EPI-, TE-, and PE- like clusters mostly overlapped with EPI, TE, and PE counterparts from blastocysts (Fig. 3c). The expression of overlapped differentially expressed genes (DEGs) between three lineages was shown in heat map (Fig. 3e; Supplementary Table S1). Overall, our scRNA-seq analysis revealed similarities between the transcriptome landscape of EPS-blastoids and early blastocysts and confirmed that, by day 6, human blastoids contained the three cell lineages found in blastocysts.

### Human EPS-blastoids can develop postimplantation embryonic structures

**Fig. 4:**
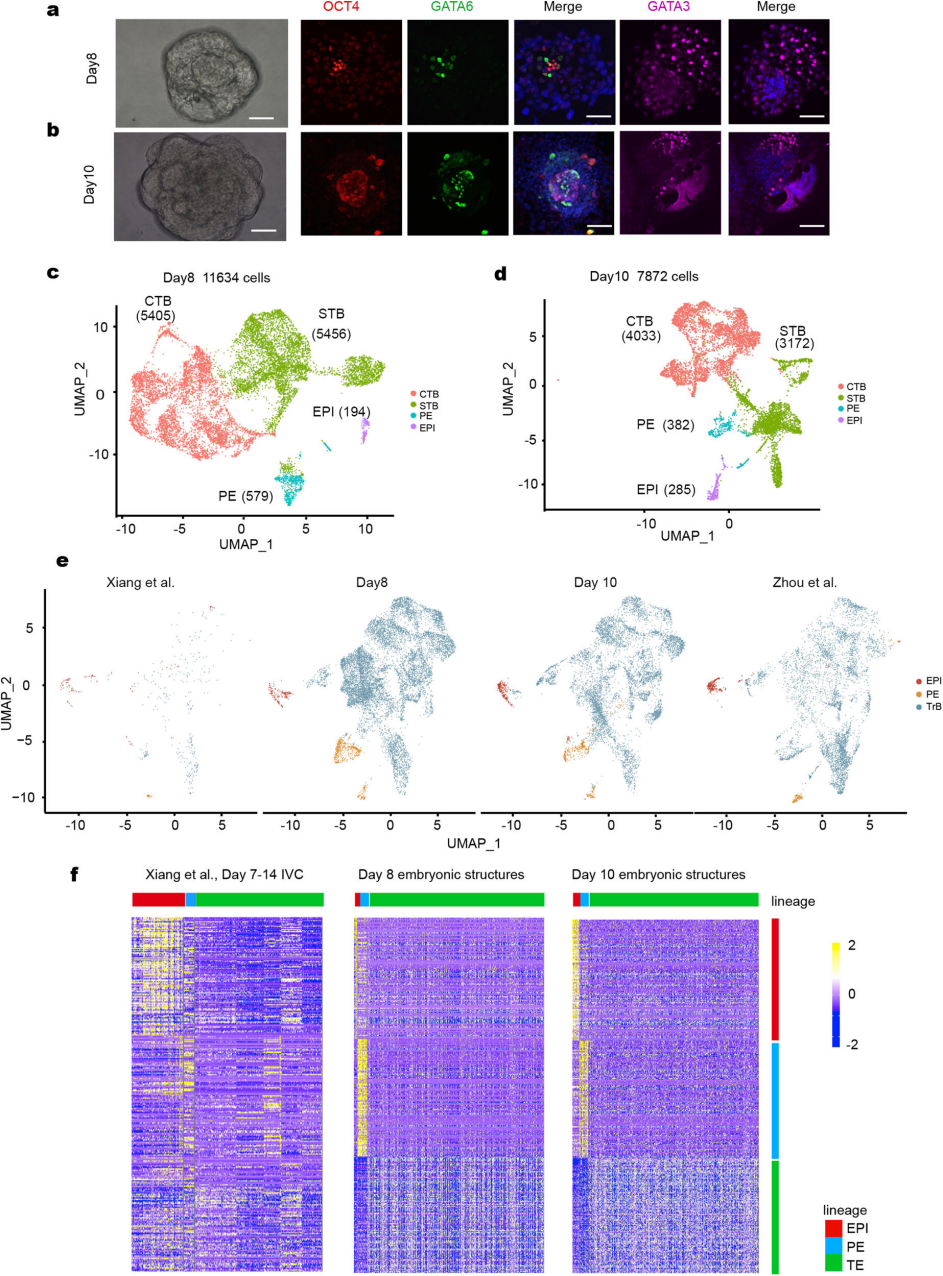
Landscape of transcriptome in human embryo-like structures from blastoids on day 8 and day 10. **a**–**d** Immunofluorescence staining of EPI marker OCT4 and PE marker GATA6 in Day 8 (**a**) and Day 10 (**b**) embryonic structures. A UMAP plot analysis revealed 5 clusters in embryonic structures on day 8 (**c**) and day 10 (**d**), identified with assigned cluster names. **e** UMAP projection of integrated datasets showing our results of embryonic structures on day 8, day 10, and previous studies^32,33^ (data of 7–14 d.p.f.). **f** Heatmap of lineage-specific genes overlapped between a previous study^32^ (data of 7–14 d.p.f. embryos) and this study on days 8 and 10.

To test whether human EPS-blastoids could undergo postimplantation morphogenesis, we kept culturing day 6 blastoids within 2–4 days (hereafter referred to as day 8 and day 10 embryonic structures) using a previously established in vitro culture (IVC) system, which needed matrigel and modified IVC1/2 medium to mimic blastocyst implantation^30,31^. On day 8, GATA6-positive cells encircled the OCT4-positive cells (Fig. 4a). On day 10, the localization patterns for OCT4 and GATA6 resembled those on day 8, except for an increased number of cells within the day 10 embryonic structures (Fig. 4b). Moreover, GATA3 positive cells were distributed around the postimplantation structure (Fig. 4a, b).

We then performed scRNA-seq analysis using day 8 (40) and day 10 (20) human embryonic structures. After quality control and filtering, 11,634 single cells from day 8 and 7872 single cells from day 10 were further analyzed. The human placenta consists of three major TrB subpopulations: CTBs, STBs, and EVTs. UMAP analysis by Seurat revealed that the primary clusters could be identified as EPI, PE, TrB (CTBs and STBs) on day 8 (Fig. 4c, Supplementary Fig. S7b), and EPI, PE, TrB (CTBs and STBs) on day 10 (Fig. 4d, Supplementary Fig. S8b). This is based on the expression of 16 representative marker genes respectively (Supplementary Fig. S6a, S7a).

To reveal similarities and differences between EPS embryonic structures and natural blastocysts subjected to IVC, we compared our results of EPS embryonic structures from days 8 and 10 with data acquired from 7 to 14 d.p.f IVC embryos^32,33^. A total of 155, 263, and 78 genes, specific for our ICM, PE, and TB clusters (day 8) overlapped with Xiang’s analyses of 7–14 d.p.f IVC embryos (Supplementary S7c; Table S1). Similarly, comparison of day 10 embryonic structures and 7–14 d.p.f. IVC embryos revealed 175, 157, and 40 genes that overlapped with our EPI, PE, and TB clusters (Supplementary Fig. S8c; Table S1), respectively. UMAP analysis revealed a concordance between cells of embryonic structures on days 8 and 10 and 7–14 d.p.f IVC embryos (Fig. 4e, Supplementary Fig. S8d). Moreover, heat map showed that our EPS embryonic structures (days 8 and 10) and 7–14 d.p.f IVC embryos had a similar transcriptional profile (Fig. 4f). In conclusion, embryo-like structures cultured from EPS-blastoids in vitro (days 8 or 10) resembled natural human embryos in terms of their single-cell transcriptome landscape in a certain degree, performing their potential in modeling human early postimplantation development.

## Discussion

Mouse EPS cells can be induced to form blastocyst-like structures (blastoids)^18,19^. Because of the significant differences between mouse and human developmental processes, it is thought that the generation of human blastoids may be more challenging. Indeed, applying the modified mouse culture system to human EPS cells failed to generate blastoids. To overcome this obstacle, we developed a 3D, two-step induction system for generating blastoids from human EPS cells. In our two-step induction system, we first exposed EPS cells to BMP4 for 3 days to induce TE-like cells formation. These TE-like cells were then mixed with EPS cells to generate EPS-blastoids. We found that TE-like cells expressed early-, mid-, and late-TE cell markers, which enhanced their subsequent developmental potential. Human EPS-blastoids were similar to human blastocysts of the same stage based on both morphology and cell lineage analysis. The latter conclusion was based on immunofluorescence and scRNA-seq analyses. The efficiency of EPS-blastoids was lower than seen for mouse blastoids (1.9% vs 15% in the human and mouse systems, respectively). One potential reason for this difference is the difficulty in maintaining stemness and pluripotency of human EPS cells in our differentiation system. The human EPS cells were difficult to maintain as dome-shaped colonies during the cultivation and contained differentiated cells that could disturb blastoids formation. Other reasons include differences between human and mouse early embryonic development and differences between the mouse and human embryo culture system. Culture medium is an important component for inducing blastoids. The efficiency of the culture system for human embryos is not as robust as the mouse system. Up to 90% of mouse embryos can develop to blastocysts in vitro, whereas only 50% of human embryos reach the blastocyst stage. The efficiency of PE lineage derivation is relatively lower than EPI and TE lineage. There are some possible reasons: First, there is no mature PE derivation protocol now, which is hard to induced EPS cells to classical PE cells; Second, the PE lineage grows mature slower than EPI and TE lineage. The PE markers often are shown express in TE cells, which is difficult to identify the right PE pattern and calculate the percentage of right phenotype. Also, the comparisons of our scRNA-seq data with previous data suggested that the human two-step blastoid differentiation system must be further optimized to achieve a potential model system for studying the human blastocyst. Moreover, it is few transcriptome data-sets available for human blastocysts and blastoids using the similar system of scRNA-seq, therefore, further detailed analysis are required from early developmental stages to postimplantation stages.

Our human EPS-blastoids recapitulated to a great extent the 3D-architecture of human blastocysts and exhibited all three developmental lineages. Functionally, we could derive both PSCs and TSCs from human EPS-blastoids. More importantly, they gave rise to postimplantation embryonic structures. These observations suggested that hESC-blastoids manifest at least some functionalities of the natural human blastocyst. The ability of hEPS-blastoid to generate several types of mature TrBs like those present in the human placenta offers great promise for studying placenta disorders in the future.

In summary, we have established an in vitro system for generating human blastoids that accurately recapitulate the development of a human blastocyst. We note that during the preparation of this manuscript three studies^21–23^ reported successful generation human blastoids from human iPSCs and naive PSCs, respectively. The human blastoid models provide an alternative and potentially high-throughput platform for exploring the mechanisms of human blastocyst development and stem cell differentiation during preimplantation and postimplantation stages.

## Materials and methods

### Human samples and ethics statement

Human skin fibroblasts were isolated from chest of a female aborted fetus that were obtained with informed written consent and approval by the Third Affiliated Hospital of Guangzhou Medical University. The generation of iPSCs with donated human fibroblasts was approved by the Ethics of Third Affiliated Hospital of Guangzhou Medical University. Human blastocysts produced from in vitro fertilization for clinical purposes were got with informed written consent and approval by the Third Affiliated Hospital of Guangzhou Medical University. All procedures were approved by the Institutional Review Board of the Third Affiliated Hospital of Guangzhou Medical University (2020027), and Peking University Third Hospital (S2020022).

### Generation of human EPS cells from human PSCs

The human PSCs and EPS cells culturing conditions were as follows: 37 °C, 5% CO_2_ and saturated humidity. Human iPSCs were generated using the electroporation (4D-Nucleofector System, Lonza) of fibroblasts with episomal vectors, including pCXLE-hOCT3/4-shp53-F, pCXLE-hSK, and pCXLE-hUL as previously reported^34^. The derived iPSCs were cultured on mitomycin C-treated MEF feeder cells in human ESC medium. The human ESC medium consisted of DMEM/F12 (Thermo Fisher Scientific, 11330-032) supplemented with 20% Knockout Serum Replacement (Thermo Fisher Scientific, A3181502), 0.1 mM NEAA (nonessential amino acids, Thermo Fisher Scientific, 10828-028), 2 mM L-glutaMAX (Thermo Fisher Scientific, 35050-061), 0.1 mM β-mercaptoethanol (Thermo Fisher Scientific, 21985-023), 1% penicillin/streptomycin (Thermo Fisher Scientific, 15140-122), and 10 ng/ml recombinant bFGF (Thermo Fisher Scientific, PHG0261). Human iPSCs were converted to EPS cells referred to protocol described previously^16^. Human iPSCs were digested into single cells by TrypLE Express Enzyme (Thermo Fisher Scientific, 12604021) or Accutase (Stem Cell Technologies, #07920) and seeded to the plate with fresh ICR MEF cells. After 12 h seeding, the human ESC medium was replaced with human N2B27-LCDM medium. N2B27 basal medium was prepared as following: 240 ml DMEM/F12 (Thermo Fisher Scientific, 11330-032), 240 ml Neurobasal (Thermo Fisher Scientific, 21103-049), 2.5 ml N2 supplement (Thermo Fisher Scientific, 17502-048), 5 ml B27 supplement (Thermo Fisher Scientific, 12587-010), 1% nonessential amino acids (Thermo Fisher Scientific, 11140-050), 1% L-glutaMAX (Thermo Fisher Scientific, 35050-061), 0.1 mM β-mercaptoethanol (Thermo Fisher Scientific, 21985-023), 1% penicillin/streptomycin (Thermo Fisher Scientific, 15140-122), 5% knockout serum replacement (KSR, Thermo Fisher Scientific, A3181502). Human N2B27-LCDM medium consisted of N2B27 basal medium supplemented with 10 ng/ml human LIF (R&D Systems, 7734), 3 μM CHIR99021 (Tocris, 4423), 2 μM (S)-(+)-Dimethindene maleate (Tocris, 1425), and 2 μM minocycline hydrochloride (Selleckchem, S4226), 1 μM IWR endo-1 (Selleckchem, S7086), and 2 μM Y-27632 (Selleckchem, S1049). The N2B27-LCDM medium was changed every day. Dome-shaped colonies emerged after 3–6 days. Then the cells were dissociated and passaged to the next generation.

### In vitro 3D generation of human EPS-blastoids

The blastoids culturing conditions were as follows: 37 °C, 5% O_2_, 5% CO_2_ and saturated humidity. Human EPS cells were differentiated with BMP4 for 3–4 days. BMP4 differentiation medium was composed of N2B27 basal medium, supplemented with 25 ng/ml BMP4 (R&D Systems, 314-BP-010), 2 μM Y-27632 (Selleckchem, S1049). On day 0, the cultured EPS cells were digested into single cells by TrypLE Express (Thermo Fisher Scientific), and the MEF cells were removed by pasting twice on 0.5% gelatin (Sigma) for 15–20 min each time. The EPS cells were collected, centrifuged, and seeded into plate pretreated with Matrigel in BMP4 differentiation medium. After 3 days of differentiation, cells were dissociated into single cells with Tryple Express. Mix the BMP4-treated cells (1.0 × 10^5^ cells) and EPS cells (2.0 × 10^4^ cells) together following 5:1 ratio to total 1.2 × 10^5^ cells per well with 0.5 ml culture medium, and then seeded into one well of 24-well aggreWell400 culture plate pretreated with anti-adherence rinsing solution (Stem Cell Technologies, #07010) following instruction. The culture medium was slightly changed without disturbing aggregates in the V-shape bottom every day and aggregates were collected on day 6. The culture medium of human EPS-blastoids is composed of EPS medium and IVC1 medium in a ratio of 1.5:1 (v/v). IVC1 culture medium is composed of Advanced DMEM/F12 (Thermo Fisher Scientific, 12634-010), 20% Heat-inactivated FBS (Thermo Fisher Scientific, 30044333), 2 mM L-glutaMAX (Thermo Fisher Scientific, 35050-061), 0.5% penicillin/streptomycin (Thermo Fisher Scientific, 15140-122), 1% ITS-X (Thermo Fisher Scientific, 51500-056), 1% sodium pyruvate (Thermo Fisher Scientific), 8 nM β-estradiol (Sigma-Aldrich, E8875), 200 ng/mL progesterone (Sigma-Aldrich, P0130) and 25 μM N-acetyl-L-cysteine (Sigma-Aldrich, A7250).

### In vitro culture of EPS-blastoids for 8 and 10 days

The method of embryo extended cultured in vitro refers to the previous studies^30,31^. The blastoids were collected on day 6, and transferred to the eight-well plate (treated with Matrigel 30 min in advance), and cultured with IVC1 for 2 days. On day 8, observe whether blastoids were adherent to the bottle of the dish or not, and replace the culture medium with IVC2 for further culture if the blastoids were adherent to the well till to day 10. The composition of IVC1 medium is the same as describe in the “In vitro 3D generation of human EPS-blastoids”, IVC2 culture medium is composed of Advanced DMEM/F12, 30% Knockout serum, 2 mM L-glutaMAX, 0.5% penicillin/streptomycin, 1% ITS-X, 1% sodium pyruvate, 8 nM β-estradiol, 200 ng/mL progesterone, 2 μM Y27632 and 25 μM N-Acetyl-L-cysteine.

### Derivation of stem cells from human blastoids

To derive ES cells^28^, individual EPS-blastoid was transferred onto a MEF layer in a 96-well plate and cultured with human ESC culture medium (see above). After 2–3 days, EPS-blastoid attached to the MEF and outgrew. Then outgrowth was digested by TrypLE Express and transfered into a new MEF feeder layer. Colony was picked, digested, and seeded onto a new MEF plate for ESC derivation. To derive TS cells^29^, EPS-blastoid was seeded in a 48-well plate coated with 5 μg/ml Collagen IV (Coring, 354233) at 37 °C overnight and cultured in human TS medium (DMEM/F12 supplemented with β-mercaptoethanol (Thermo Fisher Scientific), 0.2% FBS (Thermo Fisher Scientific), 0.5% penicillin/streptomycin, 0.3% BSA, 1% ITS-X supplement, 1.5 μg/mL L-ascorbic acid (Sigma, A4403), 50 ng/mL EGF (Sigma, E5036), 2 μM CHIR99021 (Tocris, 4423), 0.5 μM A83-01 (Selleckchem, S7692), 1 μM SB431542 (Selleckchem, S1067), 0.8 mM VPA (Selleckchem, S1168) and 5 μM Y27632 (Sellleckchem). When cells reached 70% confluence, cells were digested by TrypLE Express Enzyme and transferred into a new Collagen IV-coated 96-well plate at a ratio of 1:4.

For embryoid body (EB) formation, primed PSCs at 70% confluency were gently picked using a glass needle and cultured in DMEM/F-12, GlutaMAX supplemented with 20% KSR, 1% nonessential amino acids and 55 μM β-mercaptoethanol and 2 ng/ml bFGF (Thermo Fisher Scientific) for EB formation on ultra-low-binding attachment plates (Corning), with medium replacement every other day. On day 7, the trilineage differentiation assay of primed PSCs was performed on the matrigel-coated four-well plate in EB induction media for 4–5 days.

Differentiation of TSCs into STBs and EVTs was performed and modified as previously described^29^. For the differentiation of TSCs into STBs, TSCs were seeded at a density of 4 × 10^4^ cells per well onto a four-well plate pre-coated with 2.5 μg/ml collagen IV (Sigma) and cultured in 500 μl ST differentiation medium (DMEM/F-12, L-glutaMAX supplemented with 0.3% BSA (Sigma), 4% KSR, 1% ITS-X supplement, 0.1 mM β-mercaptoethanol, 0.5% penicillin/streptomycin, 2.5 μM Y27632 and 2 μM forskolin (Selleckchem, S2449)). Medium was replaced on day 3 of differentiation, and cells were fixed on day 6. For the differentiation of TSCs into EVTs, TSCs were seeded at a density of 4 × 10^4^ cells per well onto a four-well plate pre-coated with 1 μg/ml collagen IV (Sigma) and cultured in 500 μl EVTs differentiation medium (DMEM/F-12, L-glutaMAX supplemented with 0.3% BSA (Sigma, B2064), 4% KSR, 1% ITS-X supplement, 0.1 mM β-mercaptoethanol, 0.5% penicillin/streptomycin, 2.5 μM Y27632 (Selleckchem), 100 ng/ml hNRG1 (Cell Signaling Technology, #26941), 7.5 μM A83-01 (Selleckchem) and 2% Matrigel. On day 3 of differentiation, the medium was replaced with EVT differentiation medium without hNRG1, and Matrigel (Corning) was added to a final concentration of 0.5%. On day 6 of differentiation, EVTs differentiation medium was replaced without hNRG1 and KSR, and Matrigel was added to 0.5% final concentration. The cells were cultured for an additional 2 days before analyses were performed.

### Generation of human naive PSCs from human PSCs

The cells culturing conditions were as follows: 37 °C, 5% O_2_, 5% CO_2_ and saturated humidity. PXGL naive PSCs were chemically converted from H1 ESC following a two-step conversion process as previously described. Initially, primed state PSCs in KSR/FGF were cultured for 3 days in cRM1 medium containing N2B27 basal medium, 10 ng/ml LIF, 1 μM PD0325901 (Selleckchem, S1036), and 1 mM VPA (valproic acid, Selleckchem, S1168). These cells where then propagated in PXGL naive PSCs medium containing 10 ng/ml LIF (R&D Systems, 7734), 1 μM PD0325901 (Selleckchem, S1036), 2 μM Gö6983 (Selleckchem, S2911), and 2 μM XAV939 (Selleckchem, S1180). In both conversion mediums, 10 μM Rock inhibitor Y-27632 (Selleckchem, S1049) was added to prevent cell death. Ten days after the start of the conversion process, domed-shape colonies appeared in culture. These domed-shape colonies (PXGL naive PSCs) were either picked manually or plated on 0.5% gelatin (Sigma) for 50 min to remove differentiated cells, and PXGL naive PSCs were further passaged to achieve homogenous domed-shape colonies. For BMP4 induction of PXGL naive PSCs, it is same with BMP4-treated EPS cells. BMP4 differentiation medium was composed of N2B27 basal medium, supplemented with 25 ng/ml BMP4 (R&D Systems, 314-BP-010), 2 μM Y-27632 (Selleckchem, S1049). On day 0, the cultured naive PSCs were digested into single cells by TrypLE Express, and the MEF cells were removed by pasting twice on 0.5% gelatin (Sigma) for 15–20 min each time. The naive PSCs were collected and seeded into plate pretreated with Matrigel in BMP4 differentiation medium. After 3 days of differentiation, cells were dissociated into single cells with Tryple Express. PXGL naive PSCs and EPS-derived TE-like cells were analyzed using qPCR and immunofluorescence labeling.

### Immunofluorescence labeling

The samples were fixed with 4% paraformaldehyde in phosphate-buffered saline (PBS) for 20 min at room temperature, washed three times with PBS, and permeabilized with 0.2% Triton X-100 in PBS for 15 min. After blocking with 5% BSA in PBS for 2 h at room temperature, samples were then incubated with primary antibody diluted in blocking buffer overnight at 4 °C. After primary antibody incubation, samples were washed three times with PBS containing 0.1% Tween20. Samples were washed three times with PBS containing 0.1% Tween20 and incubated with fluorescence-conjugated secondary antibodies diluted in blocking buffer at temperature for 2 h. Nuclei were stained with Hoechst 33342 (Sigma, 94403) at 1 μg/mL. Zeiss LSM 710 or 880 and Leica SP8 confocal microscopes were used for imaging. Images were processed by ZEN (Zeiss) and Fiji (ImageJ, V2.0.0) software. The primary antibodies and dilutions were following: mouse anti-OCT4 (Santa Cruz, sc5279, polyclonal, A0616, F2719, 1:200), rabbit anti-GATA6 (Cell Signaling Technology, 5851S, monoclonal, D61E4, 4, 1:1000), mouse anti-SOX2 (Abcam, ab171380, monoclonal, 20G5, GR32833, 1:200), rabbit anti-GATA2/3 (Abcam, ab182747, monoclonal, EPR17874, GR22065, 1:200), rabbit anti-CK8 (Abcam, ab53280, monoclonal, EP1628Y, 2, 1:200), mouse anti-E-cadherin (Abcam, ab1416, monoclonal, HECD-1, GR33004, 1:200), rabbit anti-TFAP2C (Cell Signaling Technology, 2320S, polyclonal, 1:200), rabbit anti-CK7 (Abcam, ab192077, monoclonal, EPR1619Y, GR325605, 1:200), mouse anti-CK7 (Invitrogen, monoclonal, OVTL12/30, MA106316, 1:200), rabbit anti-TP63 (Biotium, polyclonal, BNUB0375-100, 1:100), goat anti-CK18 (Santa Cruz, polyclonal, N-16, sc-31700, 1:100), mouse anti-GATA3 (R&D Systems, monoclonal, 634913, MAB6330, 1:100), goat anti-OCT4 (Santa Cruz, polyclonal, N-19, sc-8628, 1:100). The secondary antibodies were: Alexa Fluor 488 Goat anti-Rabbit IgG (H+L) (Thermo Fisher Scientific, A-11008), Alexa Fluor 555 Goat anti-Mouse IgG (H+L) (Cell Signaling Technology, 4409S), Alexa Fluor 647 Goat anti-Rabbit IgG (H+L) (Abcam, ab150083, GR3269213), Alexa Fluor 488 donkey anti-rabbit (H+L) (Thermo Fisher Scientific, A-21206), Alexa Fluor 568 donkey anti-mouse (H+L) (Thermo Fisher Scientific, A-10037), Alexa Fluor 647 donkey anti-goat (H+L) (Thermo Fisher Scientific, A-21447).

### Real-time quantitative PCR

Total RNA was extracted using TRIzol (Invitrogen, 15596018). RNA (2 μg) was reverse-transcribed to cDNA template using RevertAid First Strand cDNA Synthesis Kit (Thermo Fisher Scientific, K1621). qPCR was analyzed in the Applied Biosystems QuantStudio 3. The changes of genes were calculated by the comparative ΔΔCt method or ΔCt method relative to GAPDH expression. All the experiments were performed in triplicates. The primers used in this study were listed in Supplementary Table S2.

### hCG ELISA detection

The medium was collected from conditional medium of TSC-STBs. The hCG level in the medium was measured using a human CG beta (HCG beta) ELISA kit (R&D Systems, DY9034-05) according to the manufacturer’s instructions.

### RNA-sequencing and analysis

TE-like cells derived of human EPS cells that were pretreated with BMP or BAP were sequenced using paired-end reads (PE150) on a NovaSeq following directional library preparation. All bulk RNA-seq samples from this study and previously published datasets were uploaded into the online RNA-seq analysis platform by Sequentia (A.I.R) for reads mapping and statistical analysis. NOI-seq analysis was performed and PCA plot was constructed.

### Single-cell RNA-sequencing

Human EPS-blastoids were picked up by mouth pipette and washed with PBS containing 0.05% BSA. About day 6 EPS-blastoids (200), day 8 (40) and day 10 (20) human embryonic structures were collected for single-cell RNA-seq. Samples on day 6 were dissociated with enzyme mix composed of 0.5× Versene (Lonza, 17711E), 0.5× Accumax (STEMCELL Technologies, 07921) and 0.05× DNaseI (STEMCELL Technologies, 07900) at 37 °C for 30 min with agitation and terminated by 5% BSA in PBS. Samples on day 8 or 10 were dissociated with enzyme mix composed of 0.25% Trypsin (Thermo Fisher Scientific, 25200056), and 0.05× DNaseI (STEMCELL Technologies, 07900) at 37 °C for 15 min with agitation and terminated by 5% BSA in PBS. Dissociated cells were repeated pipetting and washed with PBS containing 0.05% BSA. Using single-cell 3' Library and Gel Bead Kit V3 (10× Genomics, 1000075), the cell suspension (700–1000 living cells per microliter determined by Count Star, about 10,000 dissociated cells each sample) were loaded onto the Chromium Single Cell B Chip (10× Genomics, 1000074) and processed in the Chromium single-cell controller (10× Genomics) to generate single-cell gel beads in the emulsion according to the manufacturer’s protocol. In short, single cells were suspended in PBS containing 0.04% BSA (700–1000 cells per ml). No more than 10,000 cells were added to each channel. Captured cells were lysed and the released RNA was barcoded through reverse transcription in individual GEMs^35^. Using a S1000TM Touch Thermal Cycler (Bio Rad) to reverse transcribe, the GEMs were programed at 53 °C for 45 min, followed 85 °C for 5 min, and hold at 4 °C. The cDNA was generated and then amplified, and the quality was assessed using the Agilent 4200. According to the manufacture’s instruction, Single-cell RNA-seq libraries were constructed using Single Cell 3’ Library and Gel Bead Kit V3. Finally, sequencing was performed on the Illumina Novaseq6000 sequencer with a sequencing depth of at least 60,000 reads per cell and 150 bp (PE150) paired-end reads (performed by Capital, Beijing and Novogene, Beijing).

### Analysis of single-cell RNA-sequencing (scRNA-seq) data

The Cell Ranger software was obtained from 10× Genomics website: https://support.10xgenomics.com/single-cell-gene-expression/software/downloads/latest. Alignment, filtering, barcode counting, and UMI counting were performed with Cell Ranger count module to generate feature-barcode matrix and determine clusters. Dimensionality reduction was performed using PCA and the first ten principal components were used to generate clusters by K-means algorithm and graph-based algorithm, respectively. The other clustering method is Seurat 4.0.3 (R package). The R package Seurat 4.0.3 was used to analyze feature-barcode matrix as following steps: (1) Cells whose gene numbers were less than 100, unique features count over 60,000, and mitochondrial gene ratio was more than 20% according to quality control matrix plots, were regarded as abnormal and filtered out. (2) UMI counts were normalized with SCTransform function by default settings. A nonlinear dimensionality reduction was performed using PCA, clustered with resolution setting at 0.6, and visualized by TSNE and UMAP. (3) Differentially expressed genes (DEGs) in clusters were determined by the FindAllMarkers function (Seurat 4.0.3) using a minimum upregulation of 0.25 log-fold. DEGs between lineages with uncorrected *P* values were smaller than 0.01, and log-fold change was larger than 0.25 (log2FC > 0.25) in one group were regarded as DEGs. The GO terms of DEGs in biological process were enriched using DAVID. Heat map of top DEGs between clusters was performed with DoHeatmap (Seurat 4.0.3). Then the overlapped DEGs between blastoids and blastocysts were normalized and showed in heat map by DoHeatmap (Figs. 3, 4). The basic information of the scRNA-seq profiles were available in Supplementary Table S2.

### Integrated scRNA-seq analysis

Previously published single-cell dataset from Petropoulos et al.^7^ was integrated with the day 6 blastoids dataset. Petropoulos’s 1529 cells were filtered for blastocyst cells, removing the pre-blastocyst stages to leave 1096 E5–E7 EPI, TE, and PE cells. Petropoulos’s data was processed using SCTransform. The dataset was integrated into the day 6 blastoids data after identifying integration genes (using FindIntegrationAnchors and IntegrateData) using 4000 anchor genes derived from the SCT assays. The integrated dataset of blastoids has 4000 integrated genes. UMAP were used for dimensionality reduction and FindClusters to identify clusters (resolution 0.8). Similarly, the published single-cell datasets from Xiang et al. (461 cells) and Zhou et al. (5911 cells)^32,33^ were integrated with the day 8 and day 10 blastoids datasets. Further, the published datasets from Yu et al. (4497 cells, D9 blastoids; 5i/L/A) and Liu et al. (7861 cells)^21,22^ were integrated with the day 6 blastoids and E5–E7 blastocysts datasets.

### Statistical analysis

Statistical analyses were performed with GraphPad Prism 8 software, using unpaired two-tailed Student’s *t*-tests and one-way ANOVA. All the statistical tests performed are indicated in the figure legends. The data are presented as means ± SD, and *P* < 0.05 was regarded as significant differences. For cell numbers and gene expression, the significant differences between two samples were analyzed by GraphPad Prism 8 software.

## Supplementary information

Supplementary Information

Supplementary Table S1

Supplementary Table S2

## Data Availability

scRNA-seq data have been deposited in the Gene Expression Omnibus (GEO) under accession number GSE158971 (scRNA-seq data website:). The scRNA-seq of human pre- and early postimplantation embryos (for Figs. 3, 4) are with GEO accession E-MTAB-3929^7^ and GSE136447^32^. Bulk RNA-seq of BMP-TE and BAP-TE used in extended Fig. 2 from this study can be found as followings described. First replica of PXGL-naive and PXGL_PDA83 bulk RNA-seq data were used and can be accessed at (GEO: GSE166401). iNPSC (R27A04 and R27A04), iNPSC-BAP (R26C10 and R27G02), EPS-TSC (R27DO3, R27D02, GC317, and R27C12) and iNPSC-TSC (R27B11, R27B08, R27B01, R27A12, R27B10, and R27A11) can be accessed at (https://www.ebi.ac.uk/ena/browser/view/PRJEB34037).
