# Supplementary material for: Generation of human blastocyst-like structures from pluripotent stem cells
Source: Cell Discov. 2021 Sep 7;7:81. doi: 10.1038/s41421-021-00316-8 (PMC8421367; doi:10.1038/s41421-021-00316-8)
Supplement: Supplementary file 1 — Supplementary Information [file 41421_2021_316_MOESM1_ESM.pdf]

Supplementary materials

Figure legends

Supplementary Fig. S1 BMP4 induced the specific markers of TE lineage

a Upper panel: Schematic of aggregate formation using mouse blastoid protocol (without TE-like cells mixing). Lower panel: aggregate morphology. **b, c** Immunostaining of aggregates generated using mouse blastoid protocol for EPI marker OCT4, PE marker GATA6 (b) and TE marker CK8 and GATA2/3 (c). Scale bar = 50 μm. **d** Quantification of lineage specific genes from day0 to day5 under BMP4 treatment. **e** Gene expressions of three lineage markers under BMP4 treatment from Day 0 to Day 5 analyzed by qPCR. **f** Morphology of TE-like cells in indicated induction days (day 1 to day 3). **g, h** Immunostaining of EPI marker (OCT4), PE marker (GATA6) (g) and TE marker (GATA2/3, CK8, CK7 and TFAP2C) (h) under BMP4 treatment from Day1 to Day3. Scale bar = 50 μm.

Supplementary Fig. S2 Comparison of trophectoderm progeny between EPSCs and naïve PSCs

a PCA plot comparing EPS-derived TE to published TE derivatives from naïve PSCs. **b** qPCR analysis of EPS cells and naïve PSC (PXGL)-derived TE pretreated with BMP for 3 days. The analysis included key TE marker genes and pluripotency genes. *$P<0.05$, ** $P<0.01$, *** $P<0.001$, **** $P<0.0001$, ns $P>0.05$; ns, not significant. **c** Hierarchical clustering of key TE and pluripotency genes in EPS cells, naïve PSCs,

EPS-derived TE cells and TE derivatives from naïve PSCs. **d** Immunofluorescence analysis of TE-like cells derived from EPS cells and naïve PSCs (PXGL) following BMP4 treatment. Scale bar = 50 μm.

Supplementary Fig. S3 Analysis of specific lineage markers expression

a Immunostaining of E-cadherin expression in human aggregates on day 2 (up panel) and day 3 (down panel). **b** Immunostaining of Ki-67 expression in blastoids on day 5 (up panel) and day 6 (down panel). Scale bar = 50 μm. **c-e** The proportion of OCT4 (c), CK8 (d), and GATA6 (e) positive cells in human blastoids and blastocysts. **f-h** Quantification of the percentage of human EPS aggregates with OCT4 (f), CK8 (g) and GATA6 (h) expression. Data in a, b, data are mean ± SD (n = 172 blastoids). *P< 0.05. Data in c-f, data are mean ± SD (n = 131 blastoids). *P< 0.05.

Supplementary Fig. S4 Summary of lineage specific marker expression in human blastoids under different induction system

a Immunostaining of human blastoid on day 4 for EPI marker OCT4 and PE marker GATA6. OCT4 was expressed in the center of human blastoids, GATA6 was expressed surround the OCT4 positive cells. **b-e** The blastoids with the abnormal expression or allocation of the lineage markers OCT4, SOX2, GATA2/3 and GATA6.

Supplementary Fig. S5 Derivation of human PSCs and TSCs from human EPS-blastoids

a Schematic of the experimental design for characterization of PSCs derived from blastoids. **b** Phase contrast image of PSCs derived from EPS-blastoid (blastoid-PSC). **c** Immunostaining of blastoid-PSCs for pluripotency markers OCT4, SOX2, SSEA4 and TRA-1-60, n=2. **d** Differentiation of blastoid-PSCs into ectoderm, mesoderm and endoderm lineages, n=3. **e** Schematic of the experimental design for characterization of TSCs derived from blastoids. **f** Phase contrast image of human TSCs derived from human EPS-blastoid (blastoid-TSCs). **g** Immunostaining of blastoid-TSCs for trophoblast-related markers GATA3, TFAP2C and CK8. **h** hCG protein level detected by hCG ELISA in conditioned medium collected from TSC-differentiated STBs, mean ± SD, n = 3, ***P*< 0.01. **i, j** Immunostaining of TSC-differentiated STBs (i) and EVTs (j), n=3, Scale bar =50 μm. **k** PCA analysis of transcriptome between blastoid-PSCs and blastoid-TSCs in this study and published data [25].

Supplementary Fig. S6 Lineage-specific gene expression characteristics in blastoids on day 6 by single-cell RNA-seq

a The expression of 12 representative lineage-related genes of blastoids and 4 amino-related genes on day 6 shown in UMAP plot, including EPI/ICM, PE and TE lineage. **b** Comparison of total lineage-specific genes overlapping between a previous study[7] and this study. **c** Dot plots showing the top differentially expressed genes specifically in EPI, PE or TE lineage. **d** UMAP projection of integrated datasets between our results in this study and previous studies [7,21,22].

Supplementary Fig. S7 Lineage-specific gene expression characteristics in human embryonic structures on day 8

a The expression of 12 representative lineage-specific genes of embryonic structures on day 8 shown in UMAP plot, including EPI, PE and TrB. **b** Heat map of lineage signature gene expression on day 8. **c** Comparison of total lineage-specific genes of a previous study and this study of embryonic structures on day 8.

Supplementary Fig. S8 Lineage-specific gene expression characteristics in human embryonic structures on day 10

a The expression of 12 representative lineage-specific genes of embryonic structures on day 10 shown in UMAP plot, including EPI, PE and TrB. **b** Heatmap of lineage signature genes expression on day 10. **c** Comparison of total lineage-specific genes of a previous study and this study of embryonic structures on day 10. **d** UMAP projection integrated datasets showing our results of embryonic structures on day 10 in this study and previous studies[32,33] (data of 7–14 d.p.f.).

Supplementary Table S1 The DEGs and GO enrichment of blastoids lineages on day 6, 8 and 10.

Supplementary Table S2 The basic information of the scRNA-seq profiles of blastoids on day 6, 8 and 10.

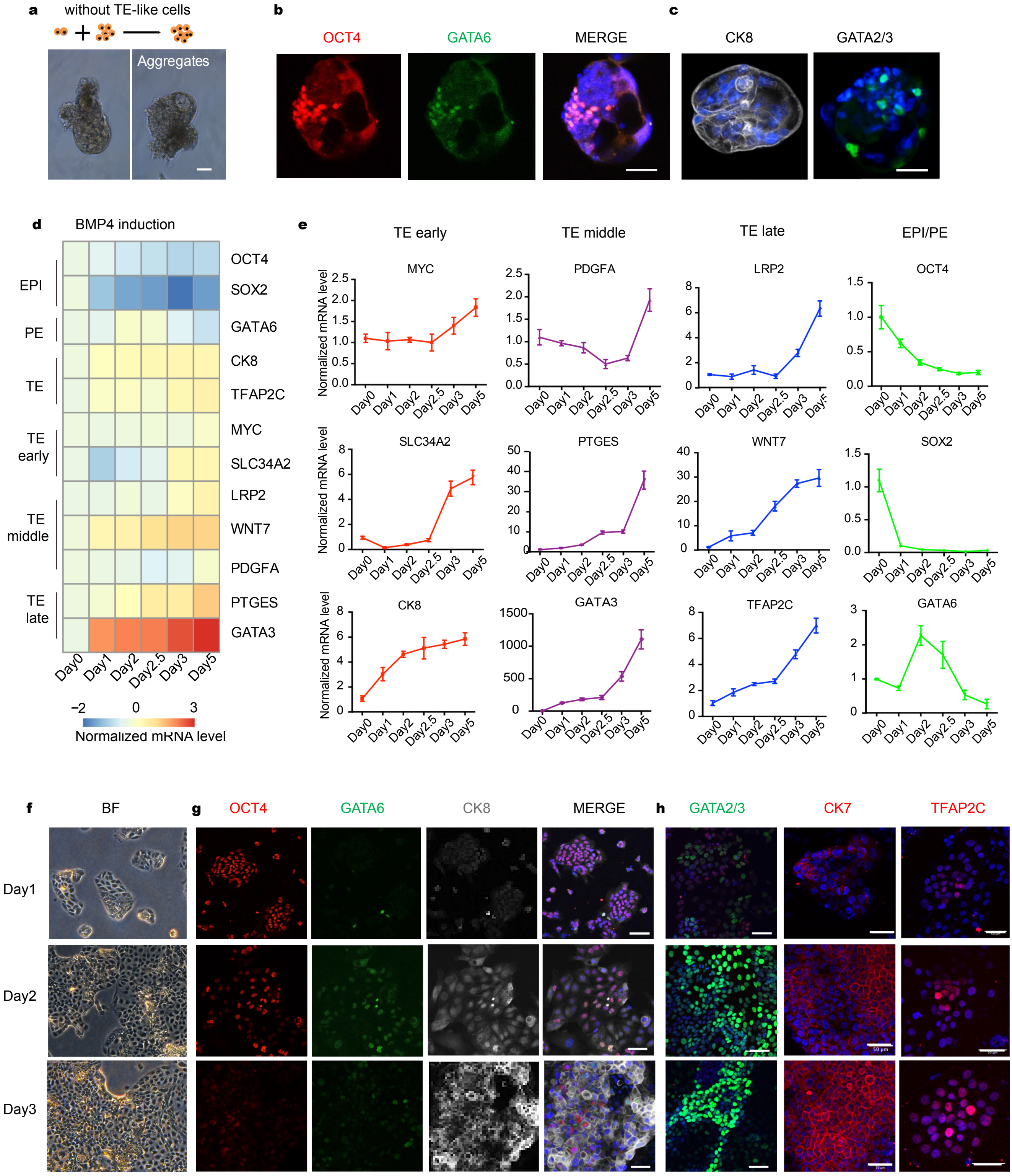

a
without TE-like cells
Aggregates
b
OCT4
GATA6
MERGE
c
CK8
GATA2/3
d
BMP4 induction
EPI
PE
TE
TE early
TE middle
TE late
OCT4
SOX2
GATA6
CK8
TFAP2C
MYC
SLC34A2
LRP2
WNT7
PDGFA
PTGES
GATA3
Day0
Day1
Day2
Day2.5
Day3
Day5
−2
0
3
Normalized mRNA level
e
TE early
TE middle
TE late
EPI/PE
Normalized mRNA level
MYC
PDGFA
LRP2
OCT4
SLC34A2
PTGES
WNT7
SOX2
CK8
GATA3
TFAP2C
GATA6
f
BF
Day1
Day2
Day3
g
OCT4
GATA6
CK8
MERGE
h
GATA2/3
CK7
TFAP2C

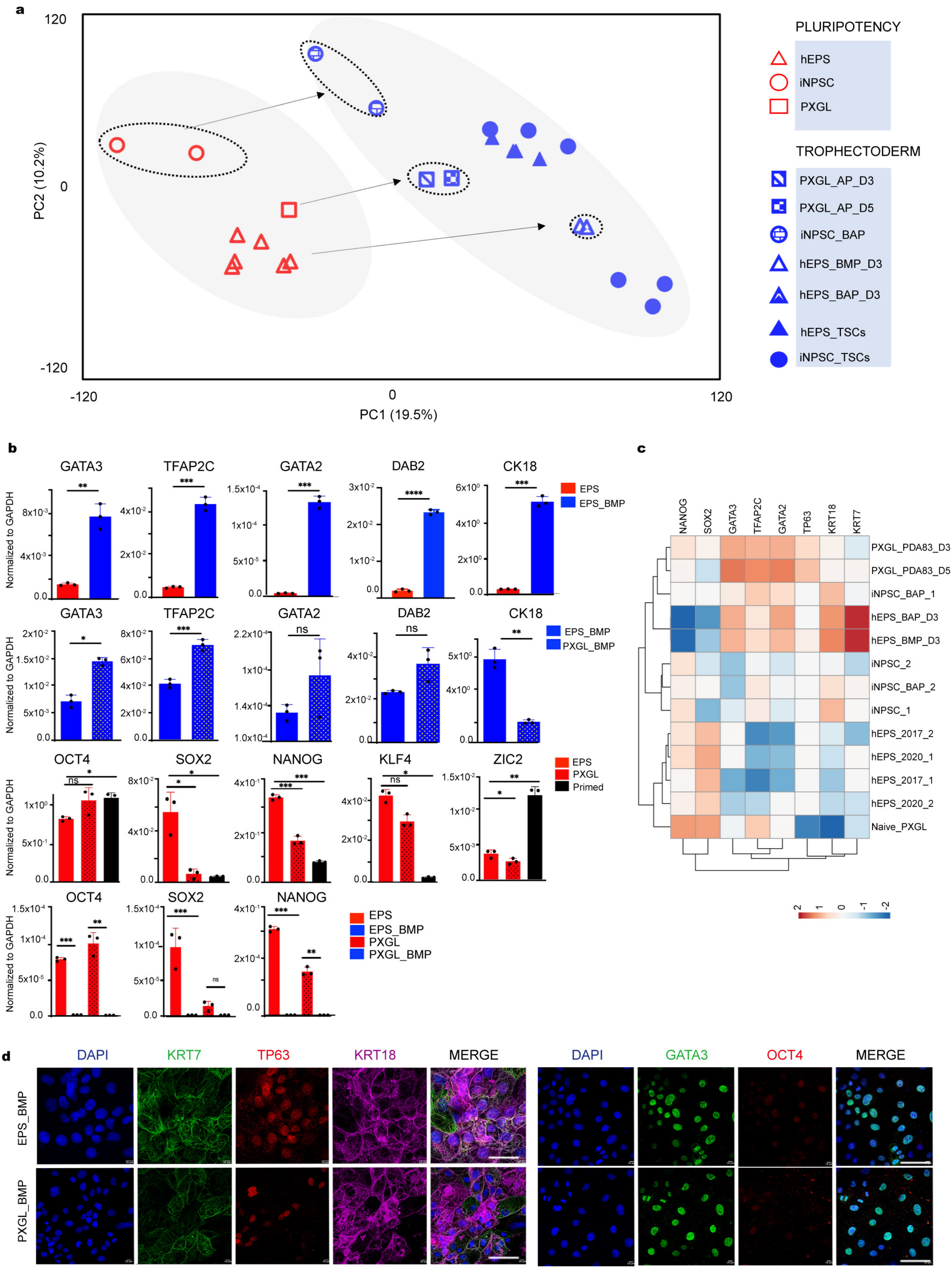
a
PC2 (10.2%)
PC1 (19.5%)
120
0
-120
PLURIPOTENCY
hEPS
iNPSC
PXGL
TROPHECTODERM
PXGL_AP_D3
PXGL_AP_D5
iNPSC_BAP
hEPS_BMP_D3
hEPS_BAP_D3
hEPS_TSCs
iNPSC_TSCs
b
GATA3
TFAP2C
GATA2
DAB2
CK18
EPS
EPS_BMP
PXGL_BMP
Normalized to GAPDH
OCT4
SOX2
NANOG
KLF4
ZIC2
PXGL
Primed
ns
c
NANOG
SOX2
GATA3
TFAP2C
GATA2
TP63
KRT18
KRT7
PXGL_PDA83_D3
PXGL_PDA83_D5
iNPSC_BAP_1
hEPS_BAP_D3
hEPS_BMP_D3
iNPSC_2
iNPSC_BAP_2
iNPSC_1
hEPS_2017_2
hEPS_2020_1
hEPS_2017_1
hEPS_2020_2
Naive_PXGL
2
1
0
-1
-2
d
DAPI
KRT7
TP63
KRT18
MERGE
GATA3
OCT4
EPS_BMP
PXGL_BMP

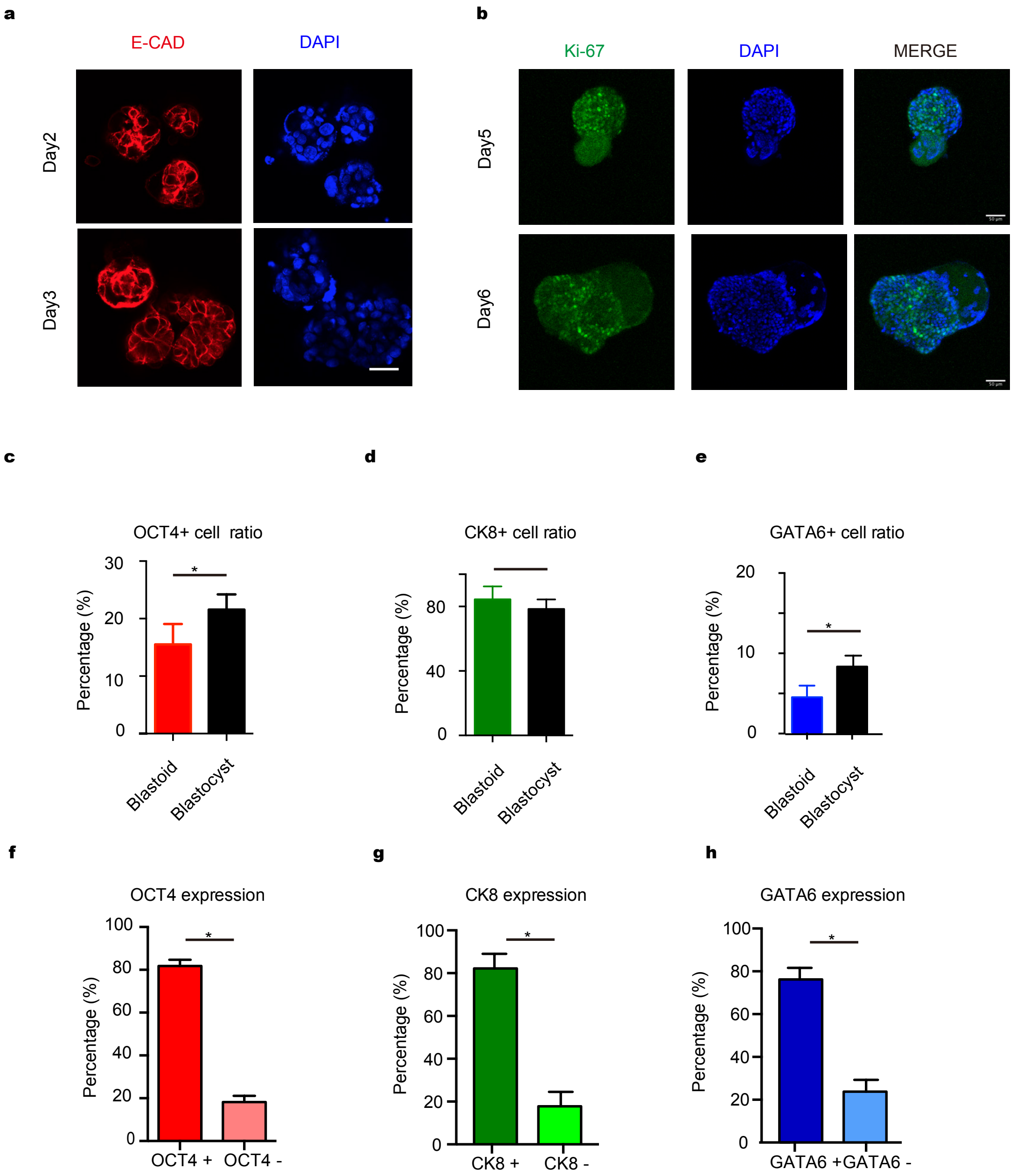

a
E-CAD
DAPI
Day2
Day3
b
Ki-67
DAPI
MERGE
Day5
Day6
c
OCT4+ cell ratio
Percentage (%)
30
20
10
0
*
Blastoid
Blastocyst
d
CK8+ cell ratio
Percentage (%)
80
40
0
Blastoid
Blastocyst
e
GATA6+ cell ratio
Percentage (%)
20
10
0
*
Blastoid
Blastocyst
f
OCT4 expression
Percentage (%)
100
80
60
40
20
0
*
OCT4 +
OCT4 -
g
CK8 expression
Percentage (%)
100
80
60
40
20
0
*
CK8 +
CK8 -
h
GATA6 expression
Percentage (%)
100
80
60
40
20
0
*
GATA6 +
GATA6 -

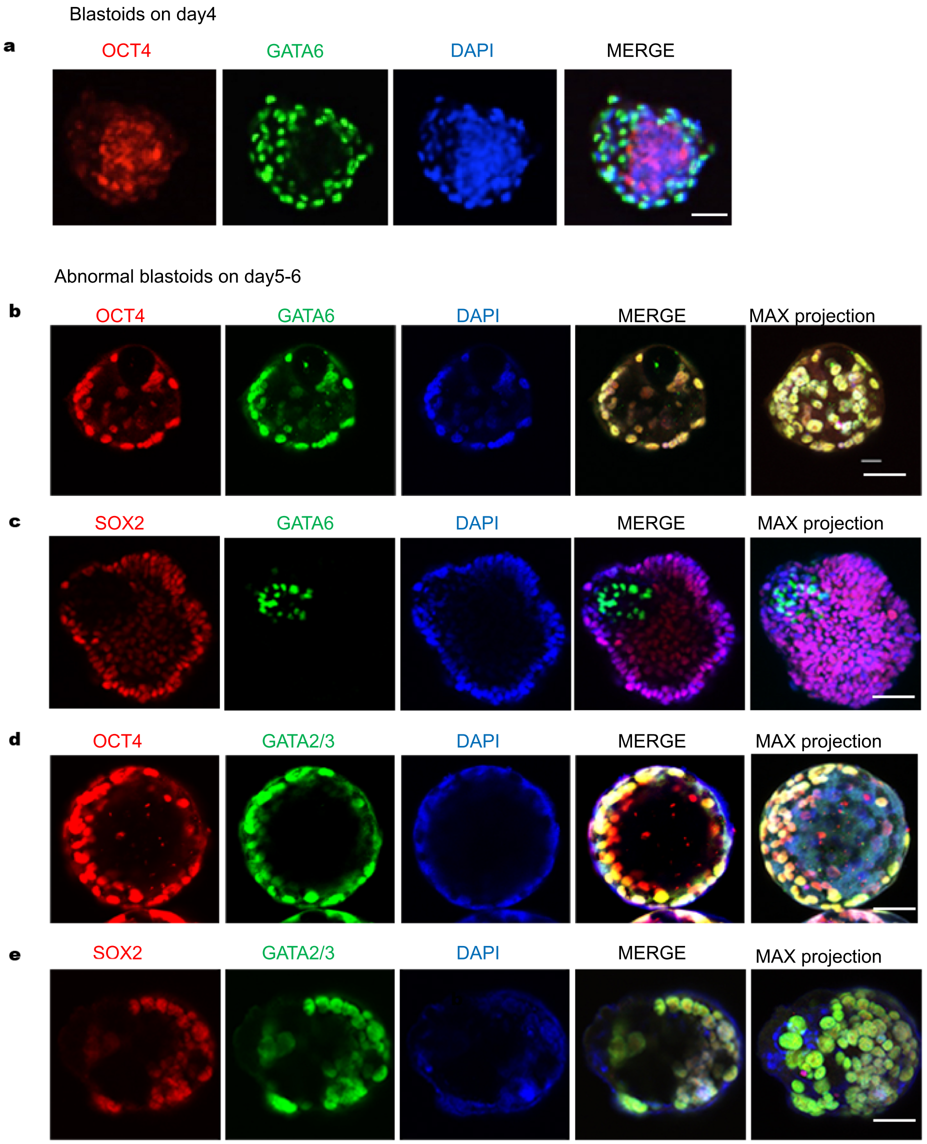

Blastoids on day4
a
OCT4
GATA6
DAPI
MERGE
Abnormal blastoids on day5-6
b
OCT4
GATA6
DAPI
MERGE
MAX projection
c
SOX2
GATA6
DAPI
MERGE
MAX projection
d
OCT4
GATA2/3
DAPI
MERGE
MAX projection
e
SOX2
GATA2/3
DAPI
MERGE
MAX projection

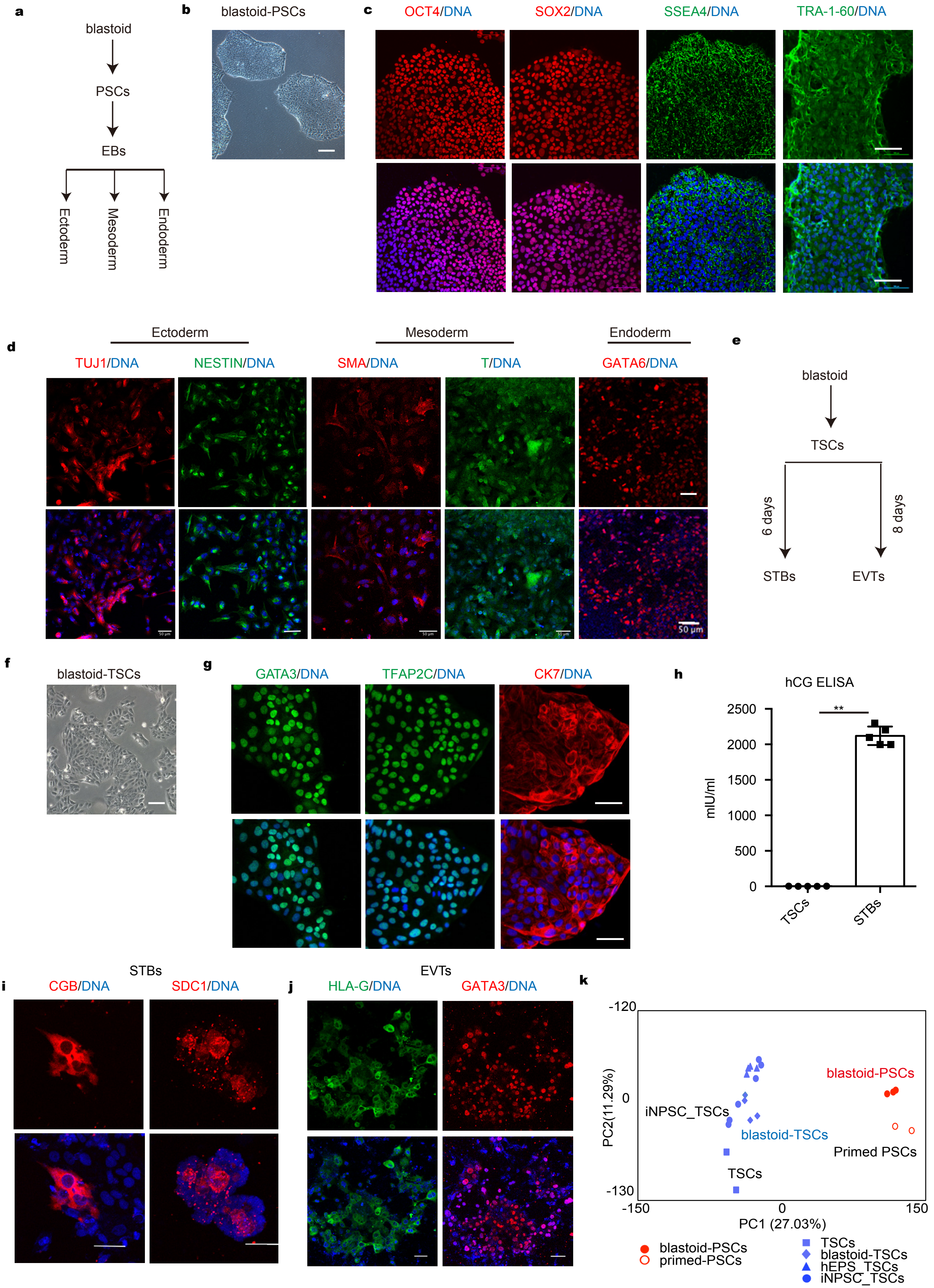
a
blastoid
PSCs
EBs
Ectoderm
Mesoderm
Endoderm
b
blastoid-PSCs
c
OCT4/DNA
SOX2/DNA
SSEA4/DNA
TRA-1-60/DNA
d
Ectoderm
Mesoderm
Endoderm
TUJ1/DNA
NESTIN/DNA
SMA/DNA
T/DNA
GATA6/DNA
50 μm
e
blastoid
TSCs
6 days
8 days
STBs
EVTs
f
blastoid-TSCs
g
GATA3/DNA
TFAP2C/DNA
CK7/DNA
h
hCG ELISA
mIU/ml
2500
2000
1500
1000
500
0
**
TSCs
STBs
i
STBs
CGB/DNA
SDC1/DNA
j
EVTs
HLA-G/DNA
GATA3/DNA
k
-120
0
-130
-150
0
150
PC2(11.29%)
PC1 (27.03%)
blastoid-PSCs
iNPSC_TSCs
blastoid-TSCs
Primed PSCs
TSCs
blastoid-PSCs
primed-PSCs
TSCs
blastoid-TSCs
hEPS_TSCs
iNPSC_TSCs

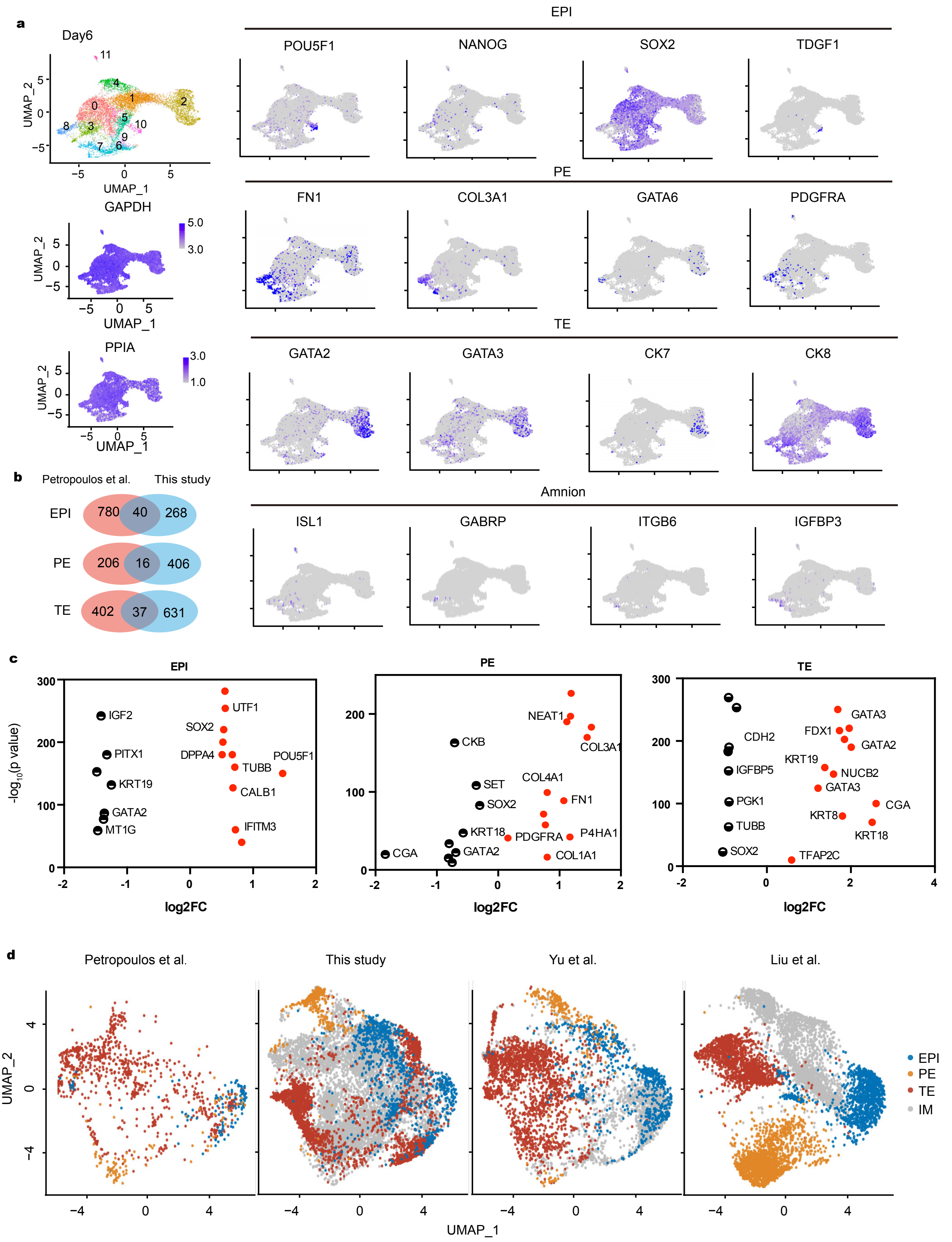
a
Day6
UMAP_1
UMAP_2
GAPDH
PPIA
EPI
POU5F1
NANOG
SOX2
TDGF1
PE
FN1
COL3A1
GATA6
PDGFRA
TE
GATA2
GATA3
CK7
CK8
Amnion
ISL1
GABRP
ITGB6
IGFBP3
b
Petropoulos et al.
This study
EPI 780 40 268
PE 206 16 406
TE 402 37 631
c
EPI
PE
TE
-log10(p value)
log2FC
IGF2
PITX1
KRT19
GATA2
MT1G
UTF1
SOX2
DPPA4
POU5F1
TUBB
CALB1
IFITM3
NEAT1
CKB
COL3A1
SET
COL4A1
SOX2
FN1
KRT18
PDGFRA
P4HA1
CGA
GATA2
COL1A1
CDH2
FDX1
GATA3
GATA2
KRT19
IGFBP5
NUCB2
GATA3
PGK1
CGA
KRT8
TUBB
KRT18
SOX2
TFAP2C
d
Petropoulos et al.
This study
Yu et al.
Liu et al.
UMAP_1
UMAP_2
EPI
PE
TE
IM

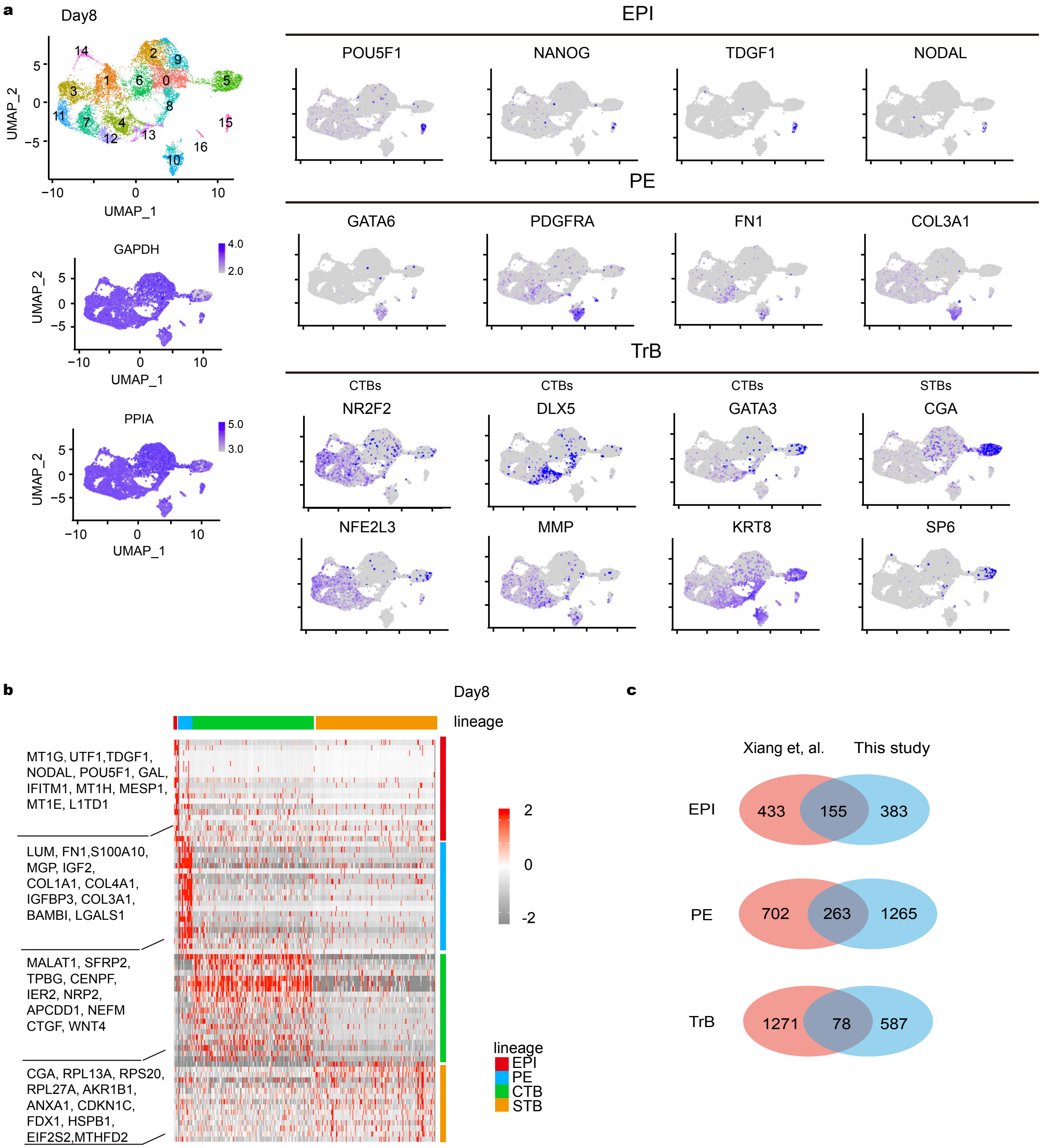

a
Day8
UMAP_2
UMAP_1
GAPDH
PPIA
EPI
POU5F1
NANOG
TDGF1
NODAL
PE
GATA6
PDGFRA
FN1
COL3A1
TrB
CTBs
NR2F2
DLX5
GATA3
STBs
CGA
NFE2L3
MMP
KRT8
SP6
b
Day8
lineage
MT1G, UTF1,TDGF1, NODAL, POU5F1, GAL, IFITM1, MT1H, MESP1, MT1E, L1TD1
LUM, FN1,S100A10, MGP, IGF2, COL1A1, COL4A1, IGFBP3, COL3A1, BAMBI, LGALS1
MALAT1, SFRP2, TPBG, CENPF, IER2, NRP2, APCDD1, NEFM CTGF, WNT4
CGA, RPL13A, RPS20, RPL27A, AKR1B1, ANXA1, CDKN1C, FDX1, HSPB1, EIF2S2,MTHFD2
lineage
EPI
PE
CTB
STB
c
Xiang et, al.
This study
EPI 433 155 383
PE 702 263 1265
TrB 1271 78 587

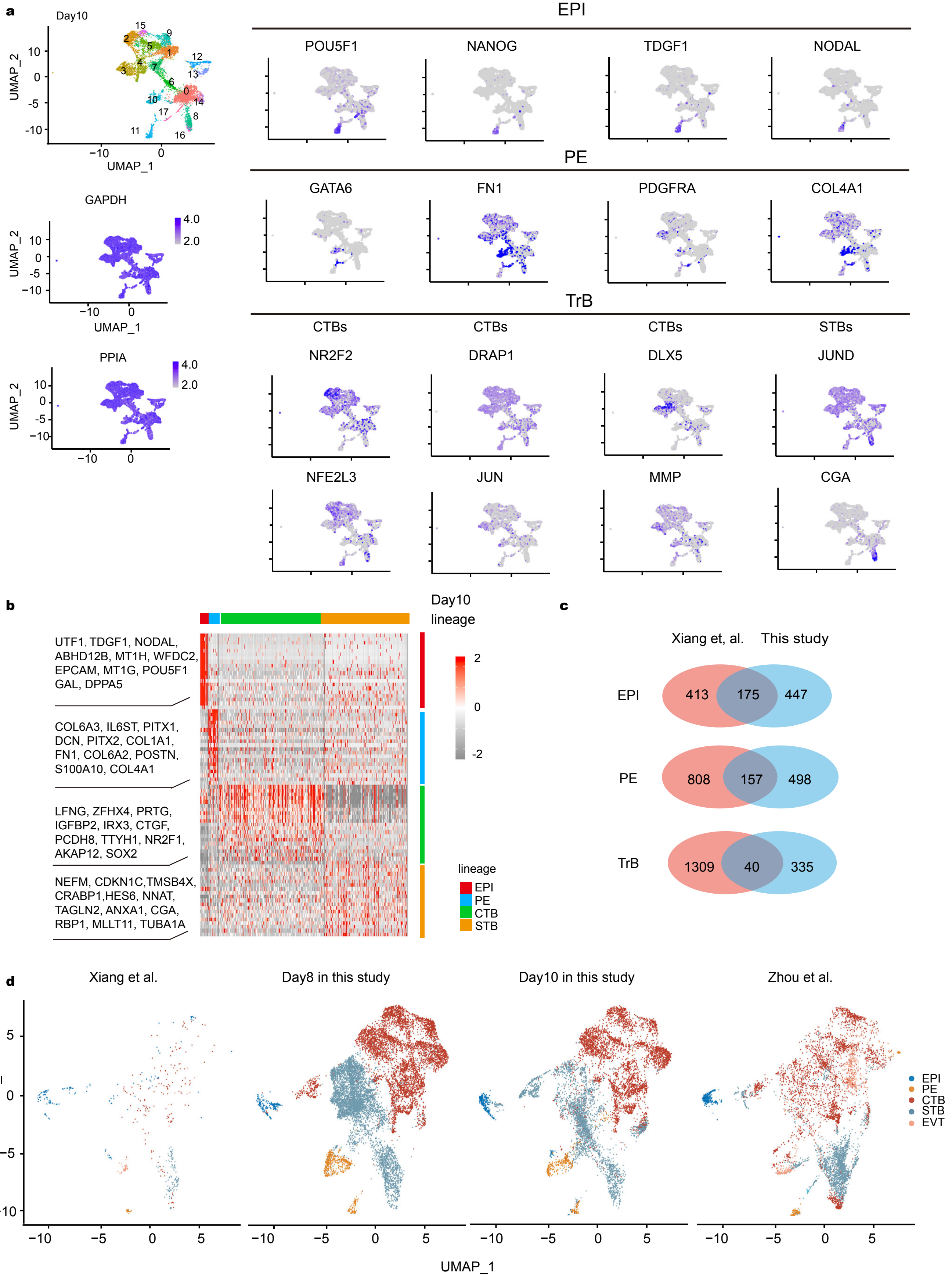
a
Day10
UMAP_2
UMAP_1
EPI
POU5F1
NANOG
TDGF1
NODAL
PE
GATA6
FN1
PDGFRA
COL4A1
TrB
CTBs
CTBs
CTBs
STBs
NR2F2
DRAP1
DLX5
JUND
NFE2L3
JUN
MMP
CGA
GAPDH
PPIA
4.0
2.0
b
Day10
lineage
UTF1, TDGF1, NODAL, ABHD12B, MT1H, WFDC2, EPCAM, MT1G, POU5F1 GAL, DPPA5
COL6A3, IL6ST, PITX1, DCN, PITX2, COL1A1, FN1, COL6A2, POSTN, S100A10, COL4A1
LFNG, ZFHX4, PRTG, IGFBP2, IRX3, CTGF, PCDH8, TTYH1, NR2F1, AKAP12, SOX2
NEFM, CDKN1C,TMSB4X, CRABP1,HES6, NNAT, TAGLN2, ANXA1, CGA, RBP1, MLLT11, TUBA1A
2
0
-2
lineage
EPI
PE
CTB
STB
c
Xiang et, al.
This study
EPI
413
175
447
PE
808
157
498
TrB
1309
40
335
d
Xiang et al.
Day8 in this study
Day10 in this study
Zhou et al.
UMAP_2
UMAP_1
EPI
PE
CTB
STB
EVT
